# Birds adapted to cold conditions show greater changes in range size related to past climatic oscillations than temperate birds

**DOI:** 10.1038/s41598-022-14972-7

**Published:** 2022-06-25

**Authors:** Lisa Carrera, Marco Pavia, Sara Varela

**Affiliations:** 1 Dipartimento di Scienze Biologiche, Geologiche e Ambientali, University of Bologna, Via Zamboni 67, 40126 Bologna, Italy; 2 Dipartimento di Scienze della Terra, Museo di Geologia e Paleontologia, University of Torino, Via Valperga Caluso 35, 10125 Turin, Italy; 3 MAPAS Lab, Departamento de Ecoloxía e Bioloxía Animal, Universidade de Vigo, 36310 Vigo, Spain

**Keywords:** Computational models, Databases, Biogeography, Climate-change ecology, Ecological modelling, Macroecology, Palaeoecology, Biogeography, Climate-change ecology, Ecological modelling, Macroecology, Palaeontology, Computational biology and bioinformatics, Ecology, Ecology, Solid Earth sciences

## Abstract

Investigation of ecological responses of species to past climate oscillations provides crucial information to understand the effects of global warming. In this work, we investigated how past climate changes affected the distribution of six bird species with different climatic requirements and migratory behaviours in the Western Palearctic and in Africa. Species Distribution Models and Marine Isotopic Stage (MIS) 2 fossil occurrences of selected species were employed to evaluate the relation between changes in range size and species climatic tolerances. The Last Glacial Maximum (LGM) range predictions, generally well supported by the MIS 2 fossil occurrences, suggest that cold-dwelling species considerably expanded their distribution in the LGM, experiencing more pronounced net changes in range size compared to temperate species. Overall, the thermal niche proves to be a key ecological trait for explaining the impact of climate change in species distributions. Thermal niche is linked to range size variations due to climatic oscillations, with cold-adapted species currently suffering a more striking range reduction compared to temperate species. This work also supports the persistence of Afro-Palearctic migrations during the LGM due to the presence of climatically suitable wintering areas in Africa even during glacial maxima.

## Introduction

The alternation of Pleistocene glacial and interglacial periods caused cyclic expansions, contractions and shifts of geographic ranges of species^1^, including bird species, shaping their current genetic structure and diversity^2–7^. As for land-based ecosystems, these range shifts are linked to latitudinal and altitudinal shift of biomes and vegetation zones in response to climate oscillations^8–10^. For instance, during the Last Glacial Maximum (LGM: 19–26,500 years ago)^11^, Mediterranean Europe was a climatic refugia for warm-adapted bird species, whereas the cold-adapted species were more widespread^12–14^.

Investigation of adaptive responses and distributional shifts of bird species to past climate changes provide crucial information to understand present and future effects of global warming and to adopt suitable conservation strategies. Past geographic distributions can be reconstructed with the help of the fossil record. GIS paleoclimatic layers and mathematical tools, such as Species Distribution Models (SDM), allow to project the current climatic requirements of species onto different past climatic scenarios, generating predictions of past distribution of species that are basically envelopes of climatic suitability, assuming niche conservatism over time^15–19^. Climatic envelopes are increasingly used to explore the potential distribution of species in the past and test evolutionary and biogeographical hypotheses. They are often integrated with molecular data to reconstruct detailed phylogeography and past population history of species^7,20–25^^.^ Many works explore LGM species population dynamics^23,26–31^; some of these are exclusively focused on bird taxa, aiming to clarify population dynamics in the refugia during climatic extremes, test niche stability, identify climate threats and effects to optimize current conservation efforts^20–22,24,32–42^. Among these, very few use the information provided by the fossil record to assess palaeobiogeographic hypotheses or to calibrate (or validate) the LGM predictive models^20,22,39,41^ and only two include the modelling of LGM wintering grounds of Afro-Palearctic migrants^20,39^.

The relationship between changes in range size (as effect of climate changes) and species thermal niche (climatic tolerance) has been theoretically investigated in mammalian species in the frame of past climatic oscillations, linking range shifts in Europe to niche optimum (cold-warm), and not to niche breadth^43^. In birds, this relationship has been investigated only for recent times’ global warming, showing that cold-dwelling species have contracted more their range size than warm-dwelling species with current climate change^44^. The Species Thermal Index (STI), i.e., the average temperature experienced by a species across its distribution (see section *Present distribution of the selected species, fossil occurrences, climatic data and Species Thermal Indexes* in Methods summary*)*, and other thermal indexes are used as a proxy to estimate the thermal niche of species, to evaluate the effect of climate change and predict population dynamics^43,45–53^.

Here, we will investigate the effect of past climate oscillations, in the Western Palearctic and Africa, on six bird species that represent different categories of migratory behaviour and climatic requires. Three are sedentary species (*Pyrrhocorax graculus*, *Athene noctua*, *Perdix perdix*), one is a partially nomadic/irruptive species (*Bubo scandiacus*) and two are long-distance migrants that winter in sub-Saharan Africa (*Coturnix coturnix*, *Crex crex*) (an outline of the ecology of the six species, together with their present-day distributions, is provided in the Supplementary Data S1). *Pyrrhocorax graculus* and *Bubo scandiacus* are restricted to cold climates whereas the other species are more climatic tolerant and adapted to a range of temperate climatic conditions^54–60^ (Table 1). These species have been selected also due to their abundance and frequency in the fossil record^61,62^, thus, to provide an empirical support of the LGM prediction models. Here, we will map present-day and LGM European and African distributions of the six species using Species Distribution Models (SDMs), test the LGM predictions with the distribution of Marine Isotope Stage (MIS) 2 fossil records, discuss range shifts and potential implications for species migratory behaviour. Finally, we will address the following hypothesis: are the range size variations related to the thermal niches?

**Table 1:** Species thermal indexes of the six bird species object of the work.

Species	Distribution	Chelsa variable	Min temp	Max temp	Range	Mean temp	Standard dev	Climatic tolerance
*Pyrrhocorax graculus*	Annual	BIO1 (mean annual temperature)	− 23.5	28.5	52	4.6	7.4	Cold-dwelling
*Bubo scandiacus*	Breeding + wintering	BIO1 (mean annual temperature)	− 24.3	10.1	34.4	− 5.2	6	Cold-dwelling
*Athene noctua*	Annual	BIO1 (mean annual temperature)	− 23.5	31.5	55	9.5	8.6	Temperate
*Perdix perdix*	Annual	BIO1 (mean annual temperature)	− 17.2	20.5	37.7	6.1	3.7	Temperate
*Crex crex*	Breeding	BIO 10 (mean temperature warmest quarter)	− 12.7	28.4	41.1	18.2	3.2	Temperate
*Coturnix coturnix*	Breeding	BIO 10 (mean temperature warmest quarter)	− 12.7	39.3	52	20.1	5.1	Temperate

The STI is identified with the average temperature experienced by a species across its distribution (i.e., mean temperature) and defines the climatic tolerance of species.

## Results

### Present-day projections

**Figure 1:**
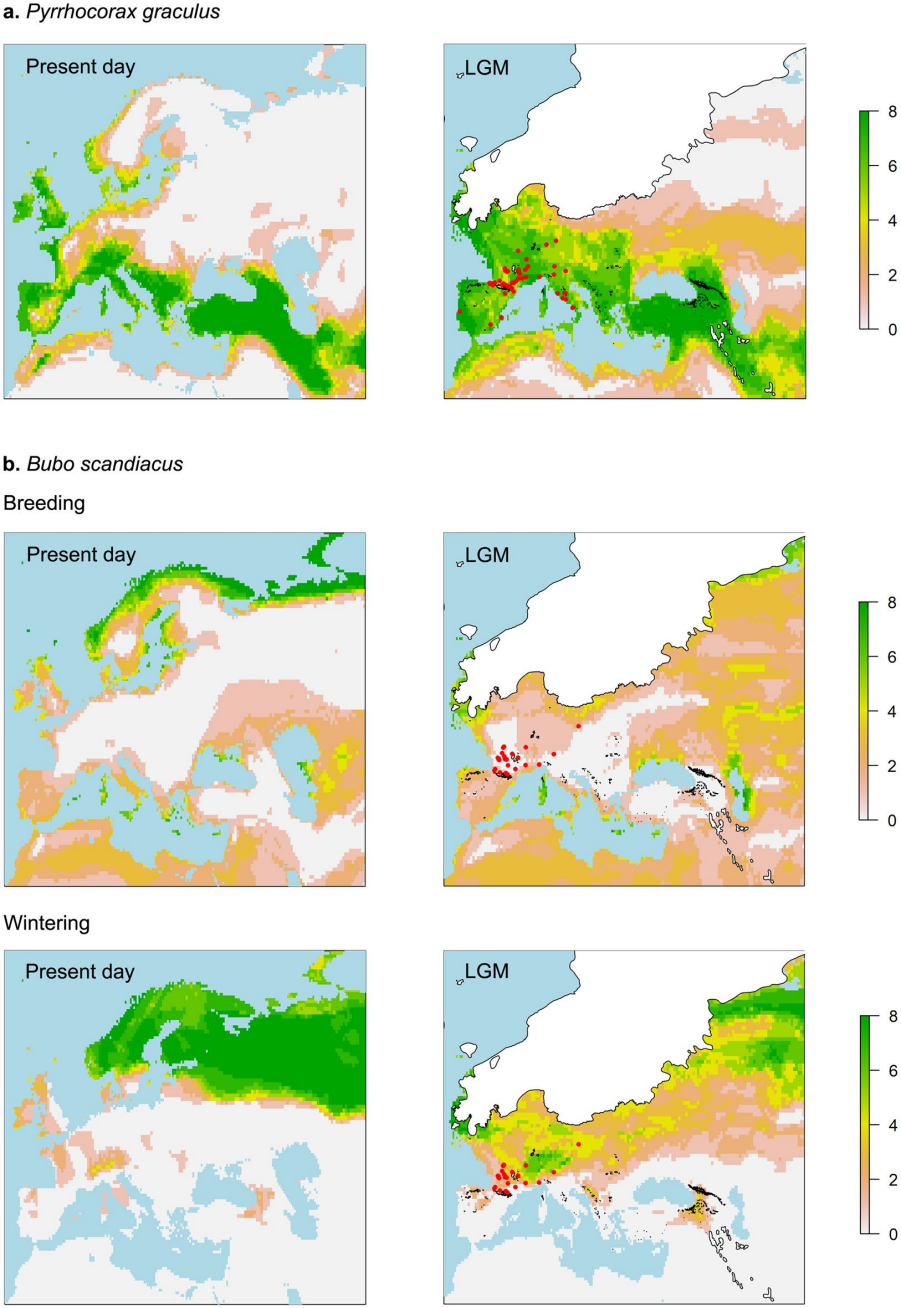
Present-day and LGM ensemble forecasts of the two cold-dwelling species *Pyrrhocorax graculus* (**a**) and *Bubo scandiacus* (**b**). The ensembles represent an averaging of all the eight projections related to the different GCMs. The values of each cell in the map range from 0 (0 out of 8 models predict the occurrence of the species in that cell, colour light grey) to 8 (all 8 models predict the occurrence of the species in that cell, colour green). The maps were created with R, version 4.0.3 (https://www.R-project.org/).

**Figure 2:**
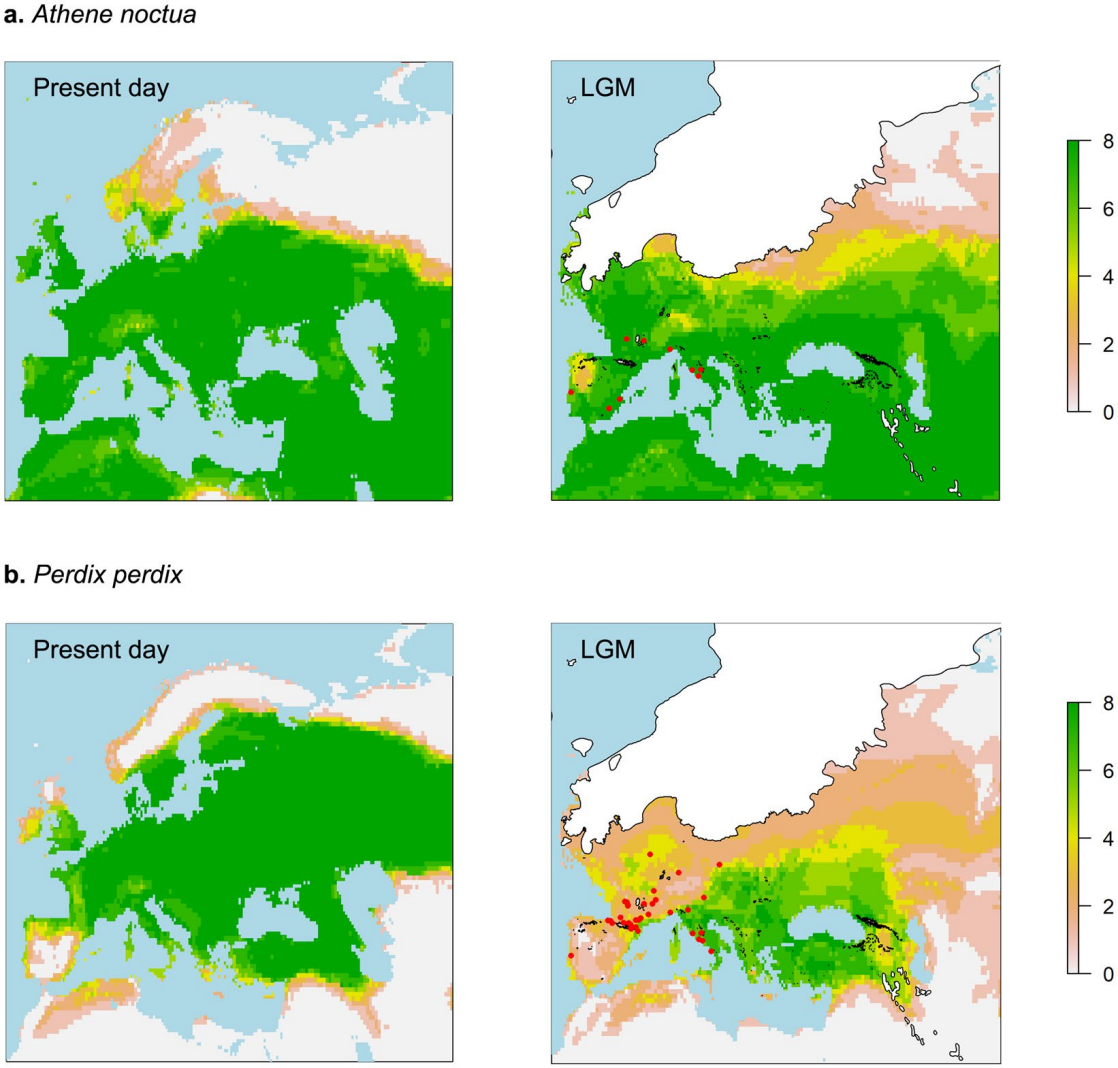
Present-day and LGM ensemble forecasts of the two resident temperate species *Athene noctua* (**a**) and *Perdix perdix* (**b**). The ensembles represent an averaging of all the eight projections related to the different GCMs. The values of each cell in the map range from 0 (0 out of 8 models predict the occurrence of the species in that cell, colour light grey) to 8 (all 8 models predict the occurrence of the species in that cell, colour green). The maps were created with R, version 4.0.3 (https://www.R-project.org/).

**Figure 3:**
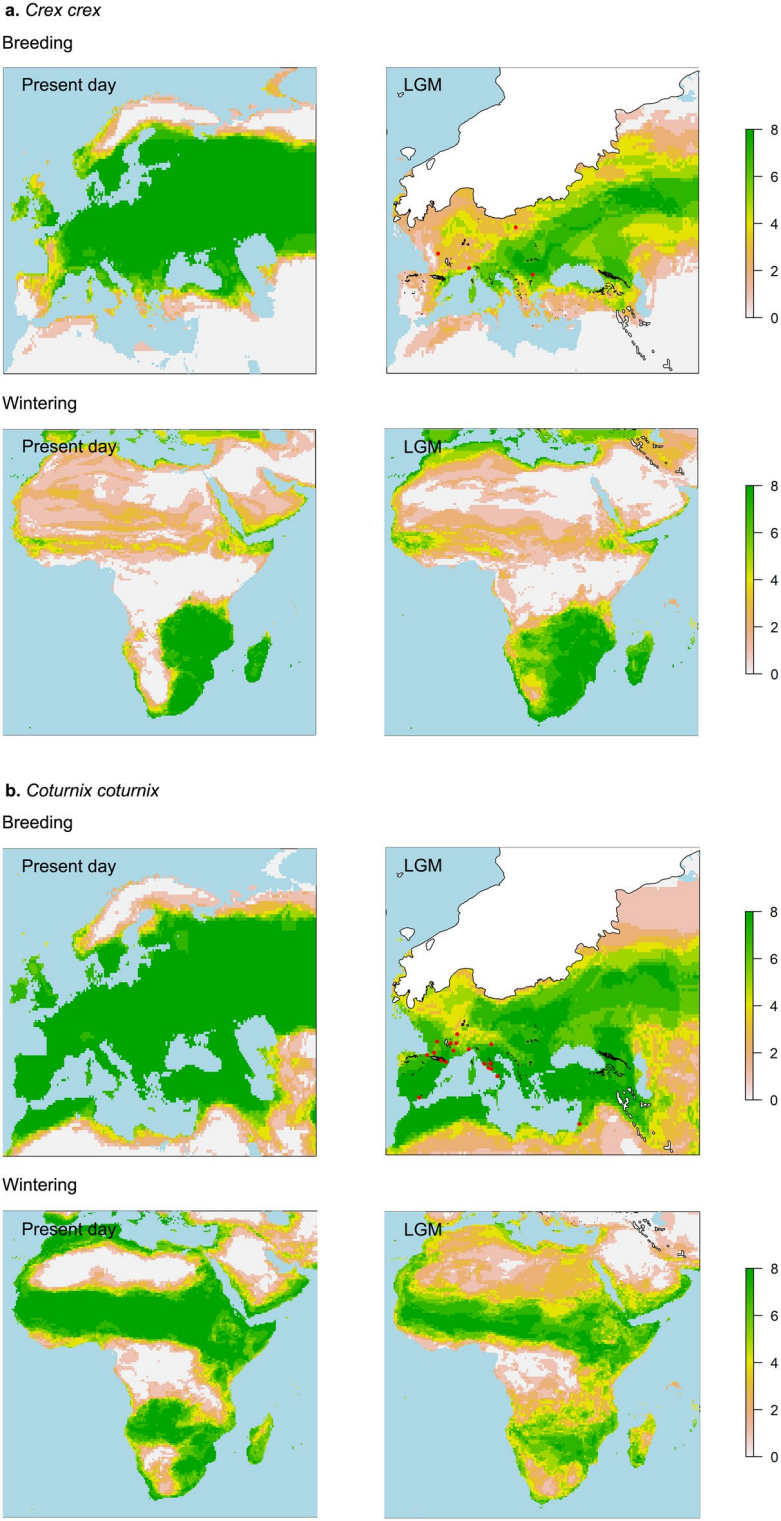
Present-day and LGM ensemble forecasts of the two migratory temperate species *Crex crex* (**a**) and *Coturnix coturnix* (**b**). The ensembles represent an averaging of all the eight projections related to the different GCMs. For each species are shown the forecasts for the breeding grounds (above) and the African wintering grounds (below). The values of each cell in the map range from 0 (0 out of 8 models predict the occurrence of the species in that cell, colour light grey) to 8 (all 8 models predict the occurrence of the species in that cell, colour green). The maps were created with R, version 4.0.3 (https://www.R-project.org/).

The model evaluation provided high AUC (Area Under the Curve) values for all the six species (see Supplementary Data S2). The projections of the models into the present-day climate are consistent with their present-day distributions (Figs. 1a, 2a, 3a; Supplementary Data S1).

### LGM climatic ensembles

*Pyrrhocorax graculus* The LGM ensemble strongly overlaps with the MIS 2 fossil occurrences of *P*. *graculus* (Fig. 1a). In the present day, this sedentary species linked to cold climates survives with a relict distribution in the mountain areas of the mid-latitudes of the Palearctic, due to Holocene climate warming. In the LGM, as witnessed by models and MIS 2 fossil records, it experienced a considerable range expansion, spreading at lower altitudes due to the downward shifts of the mountain vegetation (Fig. 1a). The lowering of the upper limit of the tree-line favoured the expansion of the mountain pastures with rocky ravines and cliff faces that this species uses for feeding and nesting.

*Bubo scandiacus* The MIS 2 fossil occurrences are not well predicted by the LGM wintering distribution ensemble neither by the breeding distribution ensemble; in any case, the LGM wintering projection predicts the fossil distribution better than the breeding projection (Fig. 1b). The models predict for the LGM a clear southward shift of both the breeding and the wintering distribution of this species, currently restricted to the arctic and boreal areas. In detail, the LGM breeding range is completely shifted towards central and southern Europe with no overlapping with the current breeding areas, probably due to the expansion of the ice cap (Fig. 1b). According to the LGM models, this species was considerably more widespread than the present day, as approximately the whole Europe satisfied the conditions of climatic suitability of this species, if we consider both the breeding and the wintering projections.

*Athene noctua* The LGM predicted distribution of this species is well supported by the MIS 2 fossil occurrences, that are limited to Southern Europe (Italy, Spain, Portugal and Southern France) (Fig. 2a). A southward shift of the northern edges of the range due to the expansion of the ice cap is predicted by the LGM climatic envelopes, causing a reduction of the distribution area compared to the present distribution (Fig. 2a). The range reduction during LGM could have led to isolation of the different populations in the Mediterranean refugia (as during other glacial maxima^6^) as this species is best adapted to temperate and warm climates and has a limited dispersal capacity^60,63,64^.

*Perdix perdix* The MIS 2 fossil occurrences of this species are not very well predicted by the LGM ensemble (Fig. 2b). The models, together with the fossil occurrences, indicate that this sedentary species linked to open environments could have undergone a southward shift of the northern edges of its current distribution during the LGM, causing a slight range reduction compared to the present one (Fig. 2b). This is possibly due to the expansion of the ice cap and the consequent presence of unsuitable climatic and environmental conditions at the northern latitudes. This species proves to be tolerant to cold climatic conditions as, even during MIS 2, it seems to persist up to latitude 50° N.

*Crex crex* The LGM breeding ensemble is only partially supported by the MIS 2 fossil occurrences, that are very limited for this species (Fig. 3a). Based on the models, this species could have undergone a reduction of the northern edges of the breeding range due to the expansion of the ice cap, causing a range reduction compared to the present one (Fig. 3a). The Late Pleistocene fossil records of this species are very rare west to the Pyrenees^61,62^ and the species is still absent from Western Europe, being mostly spread in Eastern and Central Europe. The models predict a similar distribution for the LGM. The LGM wintering African ensemble looks comparable to the current range, or even somewhat larger, but always limited to South Africa (Fig. 3a).

*Coturnix coturnix* The LGM breeding climatic envelopes support the MIS 2 fossil occurrences (Fig. 3b). The models suggest a reduction of the northern edges of the breeding ranges during the LGM compared to the present distribution, with a slight reduction of the distribution area. The LGM African wintering ensemble predicts a slight expansion of the conditions of climatic suitability in Africa during the LGM, compared to the present ones, except for the disappearing from the North African wintering areas (Fig. 3b). Nonetheless, we consider the predicted wintering presence of this species in the Sahara desert during the LGM as non-reliable, as during the LGM the Sahara was even larger than today in terms of latitudinal extent^65–68^.

## Discussion

### Consistency of LGM predictions and fossil occurrences

Generally, our LGM range maps are consistent with the fossil occurrences of the six species investigated (Figs. 1, 2, 3). However, in the cases of *Bubo scandiacus* and *Perdix perdix*, the models only partially overlap with the fossil occurrences (Figs. 1b, 2b; Supplementary Data S3). In the case of *P*. *perdix*, we assume that this could be linked to the anthropic impacts that this species suffered historically. The current populations, at least in Western Europe, are composed by a mixture of wild and farmed individuals that are released for conservation or hunting purposes. This aspect influenced boththe genetic diversity of the species, as the released individuals hybridize with the wild ones with a high risk of genetic introgressions, and the natural distribution of the species, due to the release of these commercial individuals in areas outside their "natural" climate envelope^69,70^. In addition, as this species nests on the ground in grasslands, it suffered massive declines during the XXth century due to habitat loss and degradation caused by agricultural intensification. Such an intense anthropic impact is not observed in the other investigated species. Therefore, we hypothesize that the current potential climatic niche and ecological requirements of *P*. *perdix*, modified by the anthropic impact and quantified using the present-day species distribution, might be different than the Pleistocene ones.

In the case of *B*. *scandiacus*, LGM map predictions do not overlap with the fossil occurrences especially in the case of the breeding areas. Here, our results indicate that the presence of this species in the European mid-latitudes, with a concentration in Southern France, might be explained by wintering individuals, providing interesting hints about *B*. *scandiacus* phenology during the LGM (Fig. 1b). However, this prediction does not agree with^71^, where Southern France is included in the reconstructed LGM breeding range of this species. Furthermore, the species is reported to breed at the Middle Pleistocene fossil locality of l’Escal, in Southern France, based on the presence of sub-adult bones belonging to this species^61,72,73^. The wide distribution of this species during the LGM, evidenced by its fossil records, and more in general during the Late Pleistocene, contrasts with our narrow map predictions, and leads to hypothesize that the populations of this species had a wider climatic niche in the past. One of the possible reasons for this change, besides the climate warming, is the competition with the other Palearctic large owl, *Bubo bubo*. The distribution ranges of these two species widely overlapped during the Late Pleistocene, as witnessed by their fossil occurrences^61,62^. As suggested by some authors, *B. scandiacus* may have arisen in the Early Middle Pleistocene from *Bubo ibericus* sp. nov. in Southern Europe^74^. This would explain why *B*. *scandiacus* might not have always been tightly linked to glacial conditions. However, since the last late glacial, *B*. *bubo* might have increased its populations as a consequence of the climatic amelioration, forcing *B*. *scandiacus* to move to the high latitudes^71,74^. After the LGM *B*. *scandiacus* possibly changed also some morphological and ecological traits to adapt to the environmental constraints of inhabiting high latitudes. In the present day, white plumage colour might be due to the persistent snow cover in the Arctic and the habit of day hunting during summer might be related to the hours of daylight at high latitudes. However, as suggested by some authors, during the late Pleistocene, when the species was spread at the mid-latitudes, it might have been a crepuscular/night hunter throughout the whole year, and its plumage might have been darker (brownish)^71^.

The changes in the ecological requirements of both *P*. *perdix* and *B*. *scandiacus* could explain the scarce consistency between the LGM envelopes and the fossil occurrences in the Western Palearctic (Figs. 1b, 2b).

### Range shifts

Based on the STI of the six species here investigated, *Pyrrhocorax graculus* and *Bubo scandiacus* can be defined cold-dwelling species, whereas the others are more tolerant with higher average annual mean temperatures across their distribution (Table 1). During the LGM, the expansion of the ice caps represented a major constraint for the distribution of living organisms at high latitudes. Our results show a clear latitudinal shift for *B*. *scandiacus* (Fig. 1b) and a reduction of the northern edges of the range for *Athene noctua*, *Perdix perdix*, *Crex crex* and *Coturnix coturnix* (Figs. 2, 3). In *P*. *graculus* there seems to be no latitude shift, but it is just because this species never reached the higher latitudes during postglacial expansions, that were instead reached by *B*. *scandiacus* during warm phases as the current one. An altitudinal movement is instead clear in *P*. *graculus*, with shifts towards high altitudes during the warm phases and downwards during cold phases, following the alpine meadows where this species feeds (Fig. 1a). As a reference for the European and African topography, we provided the relevant elevation maps in Supplementary Figs. S4 and S5.

The models and the fossil occurrences together suggest that striking changes in range size between the present day and the LGM have occurred in the two cold-dwelling species *B*. *scandiacus* and *P*. *graculus* (Fig. 2). The former species expanded in the whole Europe during glacial times. It is reported as far south as Gibraltar (Gorham’s Cave) in the Lateglacial^75^ and in Southern Italy during the Lateglacial (Grotta Romanelli)^76^, MIS 2 (Grotta di Cardamone)^62,77^ and MIS 3 (Ingarano)^78^. During the LGM, *P*. *graculus* followed the downward shift of the upper limit of the tree-line that, together with the retreat of the forest cover, led it to spread in a considerably larger area corresponding to approximately the whole non-glaciated Europe. The different ecological requirements of these two cold-adapted species led them to adopt different strategies to cope with warming climates such as during the Holocene: *P*. *graculus* moved towards higher elevations, whereas *B*. *scandiacus* moved towards higher latitudes, but both invariably reduced their range size, being considered “glacial relicts”^79^. These species were possibly also favored during glacial times by the expansion of open areas at the expense of forest cover, as they live in rocky areas (*P*. *graculus*) and open areas (*B*. *scandiacus*).

The models suggest, for the species adapted to temperate conditions (*A*. *noctua*, *P*. *perdix*, *C*. *crex* and *C*. *coturnix*), a slight reduction of the distribution during the LGM (compared to modern ranges), due to the southern retreat of the northern margins of their distribution owed to the expansion of the ice cap. The core of the mid-latitude distribution of these species remained unchanged throughout the last 20 ka (Figs. 2, 3). Despite a slight reduction in the size of their range due to major environmental constraints at the high latitudes, these species were possibly favored by the expansion of open areas typical of the cooler climatic phases, as they all exploit grasslands and/or steppe for their survival.

Cold-dwelling species show large changes in range size between the LGM and the present day. Thus, we can infer that the thermal niche of the Eurasian birds might be a predictive trait of the potential range size variations due to climatic oscillations^43,44^. A visual examination of our map predictions show that changes in range size between the different climatic phases does not seem to be linked to the migratory behaviour, as birds with different migratory behaviours show similar variations in the range size whereas among the resident species we observed changes in range size of different magnitude. Likewise, the extent of range variations does not even seem to be related to the breadth of the thermal niche (see “Range” in Table 1). Evidence is here provided that cold adapted species are more threatened by the current climate warming and need more effective conservation measures, as their range is already considerably contracted and will suffer further reduction in the future. We expect the same difference, in reverse, for the species that are more adapted to warm climates (extreme range reductions during glacial periods and pronounced expansions during warm phases). These aspects should be investigated in further research.

As there is no standardized methodology to generate range map estimations, these are linked to the methodological choices of researchers. The LGM climatically suitable areas of *P*. *graculus*, *C*. *crex* and *A*. *noctua* modeled, respectively, by^36,39,41^, result smaller than those proposed in this work. All the three different approaches used algorithms that risk overfitting (e.g., GAMs) or Maxent with a hard threshold selection of 10th percentile of presences (forcing range maps to be small), resulting in maps that underestimate the real behaviour of these species. Our predictions have, in general, larger suitability values for Central and Northern Europe during the LGM. We believe that our maps are closer to reality, as these three species inhabit meadows and grasslands, which were widespread during the LGM. Furthermore, the northern distribution of these species during the LGM is documented by the fossil occurrences (see Figs. 1a, 2a, 3a and Supplementary Table S6).

### LGM migrations

The LGM projections of the distribution of the two long-distance Afro-Palearctic migrants *Coturnix coturnix* and *Crex crex* indicate that climatically suitable areas existed both in Africa in the wintering grounds and in the Western Palearctic in the breeding grounds, providing further evidence for the persistence of Afro-Palearctic migration during the LGM (Fig. 3). These data agree with the view that the migratory behaviour is linked to an increase in climate seasonality and deterioration of winter conditions^80–83^. Also, our results suggest that there is no evidence of the loss of the migratory behaviour in Afro-Palearctic migrants during the LGM^39^ and that, as reported in a recent research^84^, bird migration remained an important global phenomenon throughout the last 50,000 years. Our data therefore challenge the hypothesis that the migratory behaviour is mainly a phenomenon of interglacial periods (linked to the recolonization of northern de-glaciated areas in postglacial times) that considerably reduced during glacial phases^85–88^.

The paucity of the sub-Saharan African fossil record hinders the reconstruction of the presence of the Western Palearctic breeders in Africa in the past. To date, fossil evidence of the presence of *C. coturnix* and *C. crex* in Africa during the LGM is lacking. Nevertheless, the presence of Eurasian long-distance migrants in the Pleistocene fossil record of sub-Saharan Africa, together with the absence of medullary bone in these fossils, supports the existence of the Afro-Palearctic migrations during Pleistocene^39,89^. Among the LGM fossils of *C*. *coturnix*, two are located in Gorham’s Cave (Gibraltar, Spain) and Ohalo II (Israel) (Fig. 3b), along two of the main Afro-Palearctic migration routes, possibly indicating that these routes were already used during the LGM.

## Methods summary

### Present distribution of the selected species, fossil occurrences, climatic data and species thermal indexes

The main ecological characteristics of the six species here investigated, together with their present-day distributions, are reported in the Supplementary Data S1. The data polygons representing the current distribution of the six species have been downloaded from the IUCN database^54–59^, with a single polygon for the sedentary species (*Pyrrhocorax graculus*, *Athene noctua*, *Perdix perdix*) and two polygons (one for the Palearctic breeding range and one for the wintering range) in the case of the migratory species^90^ (*Bubo scandiacus*, *Coturnix coturnix*, *Crex crex*). Based on the IUCN data^54–59^, the parts of the ranges where the species are introduced have been excluded, whereas the areas where the migratory species are resident have been merged with both wintering and breeding ranges using R, version 4.0.3 (2020-10-10). The resulting polygons are what we used as “presence” polygons (present-day distributions). As the BRT (Boosted Regression Trees) models also require “absences” polygons, the latter have been created ad-hoc for each species. “Absence” polygons were built manually using QGIS, so that they closely surround the “presence” polygons (without using any specific distance to make the polygons). In this way, the current distribution limits of the investigated species can be predicted more accurately and the potential variables limiting species ranges can be identified. So, we aimed to select a calibrating data set that was able to codify, besides presences, also the real limiting factors for these species. “Presence” and “absence” polygons used for this paper can be downloaded from https://www.dropbox.com/sh/twij289ss2gqmzk/AABOYP4Tl69x4fpJ4Zff4A8xa?dl=0.

An extensive bibliographic search allowed us to identify a total of 648 Western Palearctic sites dated to the Late Pleistocene (from 130 to 11.7 ka BP)^91,92^ that yielded fossil remains of at least one of the six species (identified with certainty to species level). We collected these fossil occurrences in a dataset. Multiple fossil occurrences of the same species from different stratigraphic units of the same deposit were counted separately due to their possible different age. Then, in order to identify those reliably dated to MIS 2 (29–14 ka BP), we thoroughly checked the age of each fossil occurrence, using the Radiocarbon Palaeolithic Europe database, v. 26^93^, which reports radiocarbon conventional ages BP, or the PACEA geo‐referenced radiocarbon database^94^. Each radiometric age has then been calibrated with OxCal 4.3 program^95^, using the IntCal13 calibration curve (95% CI) ^96^. After this check, we found 48 fossil localities dating back to MIS 2, which are the ones that we used in this paper, and we georeferenced them. We report the list of the MIS 2 fossil occurrences of the six selected species in the Supplementary Table S6. It is worth mentioning that the relative abundance of fossil records in the western part of the Western Palearctic compared to the Eastern part is related to the higher number of fossil localities (mainly Palaeolithic sites) which have been investigated in the former area and shouldn’t be regarded as an indication of the absence of the species from Eastern Europe. We also searched for African fossil records of the selected species dating back to MIS 2 in the literature, but none was found, due to the paucity of studies dealing with African fossil birds. Also, the Western Palearctic fossil occurrences of the migratory species do not necessarily indicate the breeding of those species in that specific locality, as they could also belong to individuals which were migrating. Only the finding of juvenile bones or medullary bone (calcium deposit in the bone cavity linked to egg-laying in female individuals) undoubtedly indicate the breeding of a species in a certain locality^97,98^.

We downloaded rasters of climate data for the present-day and the LGM (21 ka BP) from ecoClimate^99,100^. In this dataset, eight different general circulation models (GCMs) were available (CCSM, CNRM, GISS, FGOALS, IPSL, MPI, MRI and MIROC, with a resolution of 0.5°) from the Couple Model Intercomparison Project (CMIP5) and Paleoclimate Modeling Intercomparison Project (PMIP3) working groups. We used all the eight simulations to model the present and LGM climatic envelopes of the six species.

To calculate the STI we coupled, using R (version 4.0.3), the IUCN polygons of the species’ distribution with the CHELSA present-day climate raster and extracted the mean, median, minimum and maximum values of the Annual mean temperature experienced for each species across its distribution range, that is considered a valid estimate for the species’ thermal niche^44^. For Afro-Palearctic migratory species (*C*. *coturnix* and *C*. *crex*), the STI has been calculated only for the Palearctic breeding ranges and using the Mean temperature of the warmest quarter instead that the Annual mean temperature.

The STIs of each species are provided in Table 1. The assessment of the climatic tolerance of each species has also been based on the general ecology of the species^60^. For instance, if we consider the two cold-dwelling species, *P*. *graculus* only lives in mountain areas above the tree-line whereas *B*. *scandiacus* lives at high latitudes mostly above the polar circle. On the other hand, all the other species are spread in Mediterranean areas also at the sea level, being adapted to temperate and even warm climatic conditions. These ecological traits are well supported by the Species Thermal Indexes here calculated (Table 1), that indicate *P*. *graculus* and *B*. *scandiacus* as the two species with the lowest values of average annual mean temperature experienced across their distribution.

### Species distribution models

The present-day and the LGM climatic suitability of the six species have been modelled using BRT (Boosted Regression Trees). This is a flexible technique that allows to handle different types of predictor variables, express nonlinearities and interactions, and accommodate missing data. It consists in a boosting algorithm, that iteratively calls a regression-tree algorithm to construct a combination or ‘‘ensemble’’ of trees. Regression trees are good at selecting relevant variables and model interactions, whereas boosting combines large numbers of relatively simple tree models adaptively, overcoming the inaccuracies of single tree models and giving improved predictive performance^101–104^. All models were fitted in R, version 4.0.3 (2020-10-10), using packages Rpart (version 4.1-15)^105^ and Caret (version 6.0-88)^106^.

The models were calibrated using all the 19 climatic variables available and the nowadays presence and absence polygons for each species, randomly sampling 300 species records from the presence polygons and 300 from the absence polygons. Absence polygons (generated in QGIS) were located surrounding the presence polygons, to avoid selectiong absence data far away from the observed distribution of the species. By doing this, we aim to detect the climatic variables that are actually limiting the current distribution range of the species. The selected data sets were successively randomly split in training data (80%) and testing data (20%). The selected model settings are *lr* (learning rate) of 0.1, *tc* (tree complexity) of 1 and bag fraction of 0.5. Evaluation of the model performance was calculated with the Area Under the Curve (AUC), ranging from 0.5 (random) to 1 (prefect prediction). All the values relative to evaluation and variable importance of each model are reported in the Supplementary Data S2. Models were then projected into the eight GCMs of the LGM cold climatic scenarios and the eight GCMs relative to the present-day climatic scenarios. The outputs are maps with each cell having an index of climatic suitability between 0 (no suitability) and 1 (suitability). Successively, in order to reduce the uncertainties related to individual model projections, we averaged the eight models related to the different GCMs in two ensemble forecasts for each species, one for the present-day and one for the LGM (Figs. 1, 2, 3). In the ensemble maps, the value of each cell in the grid indicates how many models (out of eight) predict the presence of the species in that given cell. The fossil occurrences were successively plotted in the LGM ensemble maps to test if the model outputs correctly predict the fossil distribution of the different species during the LGM. The values of the cells of the ensemble corresponding to each fossil occurrence have been reported in the Supplementary Data S3.

## Supplementary Information

Supplementary Information.

## Data Availability

All data generated or analysed during this study are included in this published article [and its supplementary information files].

## References

[1] Hewitt GM (2000). The genetic legacy of the Quaternary ice ages. Nature.

[2] Drovetski SV (2018). A test of the European Pleistocene refugial paradigm, using a Western Palaearctic endemic bird species. Proc. R. Soc. B.

[3] Hewitt GM (2011). Quaternary phylogeography: the roots of hybrid zones. Genetica.

[4] Nadachowska-Brzyska K, Li C, Smeds L, Zhang G, Ellegren H (2015). Temporal dynamics of avian populations during Pleistocene revealed by whole-genome sequences. Curr. Biol..

[5] Newton I (2003). Speciation and Biogeography of Birds.

[6] Pellegrino I (2014). Phylogeography and Pleistocene refugia of the Little Owl *Athene noctua* inferred from mtDNA sequence data. Ibis.

[7] Tietze DT (2018). Bird Species: How they Arise, Modify and Vanish.

[8] Carrera L, Pavia M, Peresani M, Romandini M (2018). Late Pleistocene fossil birds from Buso Doppio del Broion Cave (North-Eastern Italy): implications for palaeoecology, palaeoenvironment and palaeoclimate. Boll. Soc. Paleontol..

[9] Carrera L, Pavia M, Romandini M, Peresani M (2018). Avian fossil assemblages at the onset of the LGM in the eastern Alps: a palaecological contribution from the Rio Secco Cave (Italy). C. R. Palevol.

[10] Carrera L, Scarponi D, Martini F, Sarti L, Pavia M (2021). Mid-Late Pleistocene Neanderthal landscapes in southern Italy: paleoecological contributions of the avian assemblage from Grotta del Cavallo, Apulia, southern Italy. Palaeogeogr. Palaeocl..

[11] Clark PU (2009). The last glacial maximum. Science.

[12] Hampe A, Jump AS (2011). Climate relicts: past, present, future. Annu. Rev. Ecol. Evol. S..

[13] Holm SR, Svenning JC (2014). 180,000 years of climate change in Europe: avifaunal responses and vegetation implications. PLoS ONE.

[14] Sanchez Marco A (2004). Avian zoogeographical patterns during the Quaternary in the Mediterranean region and paleoclimatic interpretation. Ardeola.

[15] Elith J, Leathwick JR (2009). Species distribution models: ecological explanation and prediction across space and time. Annu. Rev. Ecol. Evol. S..

[16] Gavin DG (2014). Climate refugia: joint inference from fossil records, species distribution models and phylogeography. New Phytol..

[17] Nogués-Bravo D (2009). Predicting the past distribution of species climatic niches. Glob. Ecol. Biogeogr..

[18] Svenning JC, Fløjgaard C, Marske KA, Nogues-Bravo D, Normand S (2011). Applications of species distribution modeling to paleobiology. Quat. Sci. Rev..

[19] Varela S, Lobo JM, Hortal J (2011). Using species distribution models in paleobiogeography: a matter of data, predictors and concepts. Palaeogeogr. Palaeocl..

[20] Arcones A, Ponti R, Ferrer X, Vieites DR (2021). Pleistocene glacial cycles as drivers of allopatric differentiation in Arctic shorebirds. J. Biogeogr..

[21] Kozma R, Melsted P, Magnússon KP, Höglund J (2016). Looking into the past–the reaction of three grouse species to climate change over the last million years using whole genome sequences. Mol. Ecol..

[22] Lagerholm VK (2017). Range shifts or extinction? Ancient DNA and distribution modelling reveal past and future responses to climate warming in cold-adapted birds. Glob. Change Biol..

[23] Metcalf JL (2014). Integrating multiple lines of evidence into historical biogeography hypothesis testing: a *Bison bison* case study. Proc. R. Soc. B.

[24] Perktaş U, Peterson AT, Dyer D (2017). Integrating morphology, phylogeography, and ecological niche modeling to explore population differentiation in North African Common Chaffinches. J. Ornithol..

[25] Perktaş U, De Silva TN, Quintero E, Tavşanoğlu Ç (2019). Adding ecology into phylogeography: ecological niche models and phylogeography in tandem reveals the demographic history of the subalpine warbler complex. Bird Study.

[26] Fløjgaard C, Normand S, Skov F, Svenning JC (2009). Ice age distributions of European small mammals: insights from species distribution modelling. J. Biogeogr..

[27] Lima-Ribeiro MS, Varela S, Nogués-Bravo D, Diniz-Filho JAF (2012). Potential suitable areas of giant ground sloths dropped before its extinction in South America: the evidences from bioclimatic envelope modeling. Nat. Conserv..

[28] Lorenzen ED (2011). Species-specific responses of Late Quaternary megafauna to climate and humans. Nature.

[29] Martínez-Meyer E, Townsend Peterson A, Hargrove WW (2004). Ecological niches as stable distributional constraints on mammal species, with implications for Pleistocene extinctions and climate change projections for biodiversity. Glob. Ecol. Biogeogr..

[30] Nogués-Bravo D, Rodríguez J, Hortal J, Batra P, Araújo MB (2008). Climate change, humans, and the extinction of the woolly mammoth. PLoS Biol..

[31] Waltari E (2007). Locating Pleistocene refugia: comparing phylogeographic and ecological niche model predictions. PLoS ONE.

[32] Barrientos R (2014). Refugia, colonization and diversification of an arid-adapted bird: coincident patterns between genetic data and ecological niche modelling. Mol. Ecol..

[33] Huntley B, Green RE, Watson RT, Cade TJ, Fuller M, Hunt G, Potapov E (2011). Bioclimatic models of the distributions of Gyrfalcons and ptarmigan. Gyrfalcons and Ptarmigan in a Changing World.

[34] Huntley B, Allen JRM, Barnard P, Collingham YC, Holliday PR (2013). Species distribution models indicate contrasting late-Quaternary histories for Southern and Northern Hemisphere bird species. Glob. Ecol. Biogeogr..

[35] Kiss O (2020). Past and future climate-driven shifts in the distribution of a warm-adapted bird species, the European Roller *Coracias garrulus*. Bird Study.

[36] Koparde P, Mehta P, Mukherjee S, Robin VV (2019). Quaternary climatic fluctuations and resulting climatically suitable areas for Eurasian owlets. Ecol. Evol..

[37] Peterson AT, Ammann CM (2013). Global patterns of connectivity and isolation of populations of forest bird species in the late Pleistocene. Glob. Ecol. Biogeogr..

[38] Peterson AT, Martínez-Meyer E, González-Salazar C (2004). Reconstructing the Pleistocene geography of the *Aphelocoma* jays (Corvidae). Divers. Distrib..

[39] Ponti R, Arcones A, Ferrer X, Vieites DR (2020). Lack of evidence of a Pleistocene migratory switch in current bird long-distance migrants between Eurasia and Africa. J. Biogeogr..

[40] Ruegg KC, Hijmans RJ, Moritz C (2006). Climate change and the origin of migratory pathways in the Swainson's thrush *Catharus ustulatus*. J. Biogeogr..

[41] Smith SE, Gregory RD, Anderson BJ, Thomas CD (2013). The past, present and potential future distributions of cold-adapted bird species. Divers. Distrib..

[42] Sutton LJ (2021). Geographic range estimates and environmental requirements for the harpy eagle derived from spatial models of current and past distribution. Ecol. Evol..

[43] Varela S, Lima-Ribeiro MS, Diniz-Filho JAF, Storch D (2015). Differential effects of temperature change and human impact on European Late Quaternary mammalian extinctions. Glob. Change Biol..

[44] Scridel D (2017). Thermal niche predicts recent changes in range size for bird species. Clim. Res..

[45] Barnagaud JY (2012). Relating Habitat and Climatic Niches in Birds. PLoS Biol..

[46] Devictor V, Julliard R, Jiguet F, Couvet D (2008). Birds are tracking climate warming, but not fast enough. Proc. R. Soc. Lond. [Biol.].

[47] Gaüzère P, Jiguet F, Devictor V (2015). Rapid adjustment of bird community compositions to local climatic variations and its functional consequences. Glob. Change Biol..

[48] Jiguet F, Gadot A, Julliard R, Newson S, Couvet D (2007). Climate envelope, life history traits and the resilience of birds facing global change. Glob. Change Biol..

[49] Jiguet F (2010). Bird population trends are linearly affected by climate change along species thermal ranges. Proc. R. Soc. Lond. [Biol.].

[50] Jiguet F (2010). Population trends of European common birds are predicted by characteristics of their climatic niche. Glob. Change Biol..

[51] Lindström Å, Green M, Paulson G, Smith HG, Devictor V (2013). Rapid changes in bird community composition at multiple temporal and spatial scales in response to recent climate change. Ecography.

[52] Pearce-Higgins JW, Eglington SM, Martay B, Chamberlain DE (2015). Drivers of climate change impacts on bird communities. J. Anim. Ecol..

[53] Stephens PA (2016). Consistent response of bird populations to climate change on two continents. Science.

[54] BirdLife International. Crex crex. *The IUCN Red List of Threatened Species* *2016: e.T22692543A86147127*. https://dx.doi.org/10.2305/IUCN.UK.2016-3.RLTS.T22692543A86147127.en (2016).

[55] BirdLife International. Perdix perdix. *The IUCN Red List of Threatened Species* *2016: e.T22678911A85929015*. https://dx.doi.org/10.2305/IUCN.UK.2016-3.RLTS.T22678911A85929015.en (2016).

[56] BirdLife International. Pyrrhocorax graculus. *The IUCN Red List of Threatened Species* *2016: e.T22705921A87386602*. https://dx.doi.org/10.2305/IUCN.UK.2016-3.RLTS.T22705921A87386602.en (2016).

[57] BirdLife International. Coturnix coturnix. *The IUCN Red List of Threatened Species* *2018: e.T22678944A131904485*. https://dx.doi.org/10.2305/IUCN.UK.2018-2.RLTS.T22678944A131904485.en (2018).

[58] BirdLife International. Athene noctua. *The IUCN Red List of Threatened Species 2019: e.T22689328A155470112.* https://dx.doi.org/10.2305/IUCN.UK.2019-3.RLTS.T22689328A155470112.en (2019).

[59] BirdLife International. Bubo scandiacus. *The IUCN Red List of Threatened Species 2020: e.T22689055A181375387*. https://dx.doi.org/10.2305/IUCN.UK.2020-3.RLTS.T22689055A181375387.en (2020).

[60] Cramp S (1998). The Complete Birds of the Western Palearctic on CD-ROM.

[61] Tyrberg, T. *Pleistocene Birds of the Palearctic: A Catalogue.* (Publications of the Nuttall Ornithological Club No. 27, 1998).

[62] Tyrberg, T. *Pleistocene Birds of the Palaearctic.* http://web.telia.com/~u11502098/pleistocene.pdf (2008).

[63] Pellegrino I (2015). Evidence for strong genetic structure in European populations of the little owl *Athene noctua*. J. Avian Biol..

[64] van Nieuwenhuyse D, Génot JC, Johnson DH (2008). The Little Owl: Conservation, Ecology and Behavior of Athene noctua.

[65] Dupont LM (1993). Vegetation zones in NW Africa during the Brunhes chron reconstructed from marine palynological data. Quat. Sci. Rev..

[66] Hoag C, Svenning JC (2017). African environmental change from the Pleistocene to the Anthropocene. Annu. Rev. Env. Resour..

[67] Hoelzmann P, Battarbee RW, Gasse F, Stickley CE (2004). Palaeoenvironmental changes in the arid and sub arid belt (Sahara-Sahel-Arabian Peninsula) from 150 kyr to present. Past Climate Variability Through Europe and Africa.

[68] Larrasoaña JC, Roberts AP, Rohling EJ (2013). Dynamics of green Sahara periods and their role in hominin evolution. PLoS ONE.

[69] Bech N, Novoa C, Allienne JF, Boissier J, Bro E (2020). Quantifying genetic distance between wild and captive strains of the grey partridge *Perdix perdix* in France: conservation implications. Biodivers. Conserv..

[70] Liukkonen-Anttila T, Uimaniemi L, Orell M, Lumme J (2002). Mitochondrial DNA variation and the phylogeography of the grey partridge (*Perdix perdix*) in Europe: from Pleistocene history to present day populations. J. Evolut. Biol..

[71] Potapova O (2001). Snowy owl *Nyctea scandiaca* (Aves: Strigiformes) in the Pleistocene of the Ural Mountains with notes on its ecology and distribution in the Northern Palearctic. Deinsea.

[72] Mourer-Chauviré C (1975). Les oiseaux du Pléistocène moyen et supérieur de France. Doc. Lab. Géol. Fac. Sci. Lyon.

[73] Mourer-Chauviré C, Grigson C, Clutton-Brock J (1983). Les oiseaux dans les habitats pale´olithiques: gibier des hommes ou proies des rapaces?. Animal and Archaeology: 2. Shell Middens, Fishes and Birds.

[74] Meijer HJ, Pavia M, Madurell-Malapeira J, Alba DM (2017). A revision of fossil eagle owls (Aves: Strigiformes: Bubo) from Europe and the description of a new species, *Bubo ibericus*, from Cal Guardiola (NE Iberian Peninsula). Hist. Biol..

[75] Sanchez Marco A (2018). Aves fósiles de la Península Ibérica, Canarias y Baleares: balance de los estudios realizados. Investig. Rev. PH Inst. Andal. Patrim. Hist..

[76] Sardella R (2018). Grotta Romanelli (Southern Italy, Apulia): legacies and issues in excavating a key site for the Pleistocene of the Mediterranean. Riv. Ital. Paleontol. S..

[77] Rustioni M, Ferretti MP, Mazza P, Pavia M, Varola A (2003). The vertebrate fauna from Cardamone (Apulia, southern Italy): an example of Mediterranean mammoth fauna. Deinsea.

[78] Bedetti C, Pavia M (2007). Reinterpretation of the Late Pleistocene Ingarano Cave deposit based on the fossil bird association (Apulia, South-eastern Italy). Riv. Ital. Paleontol. S..

[79] Tyrberg T (1991). Arctic, montane and steppe birds as glacial relicts in West Palearctic. Ornithol. Verh..

[80] Bruderer B, Salewski V (2008). Evolution of bird migration in a biogeographical context. J. Biogeogr..

[81] Finlayson C (2011). Avian Survivors. The History and Biogeography of Palearctic Birds.

[82] Louchart A (2008). Emergence of long distance bird migrations: a new model integrating global climate changes. Naturwissenschaften.

[83] Winger BM, Auteri GG, Pegan TM, Weeks BC (2019). A long winter for the Red Queen: rethinking the evolution of seasonal migration. Biol. Rev..

[84] Somveille M (2020). Simulation-based reconstruction of global bird migration over the past 50,000 years. Nat. Commun..

[85] Fiedler W, Berthold P, Gwinner E, Sonnenschein E (2003). Recent changes in migratory behaviour of birds: a compilation of field observations and ringing data. Avian Migration.

[86] Milá B, Smith TB, Wayne RK (2006). Postglacial population expansion drives the evolution of long-distance migration in a songbird. Evolution.

[87] Zink RM (2011). The evolution of avian migration. Biol. J. Linn. Soc..

[88] Zink RM, Gardner AS (2017). Glaciation as a migratory switch. Sci. Adv..

[89] Matthiesen DG (1990). Avian medullary bone in the fossil record, an example from the Early Pleistocene of Olduvai Gorge, Tanzania. J. Vertebr. Paleontol..

[90] Ponti R, Arcones A, Ferrer X, Vieites DR (2020). Seasonal climatic niches diverge in migratory birds. Ibis.

[91] Cohen KM, Gibbard PL (2019). Global chronostratigraphical correlation table for the last 2.7 million years, version 2019 QI-500. Quat. Int..

[92] Lisiecki LE, Raymo ME (2005). A Pliocene-Pleistocene stack of 57 globally distributed benthic δ^18^O records. Paleoceanography.

[93] Vermeersch, P. M. *Radiocarbon Palaeolithic Europe Database, Version 26*. https://ees.kuleuven.be/geography/projects/14c-palaeolithic/index.html (2019). 10.1016/j.dib.2020.105793 PMC7300123 32577447

[94] d’Errico F, Banks WE, Vanhaeren M, Laroulandie V, Langlais M (2011). PACEA geo-referenced radiocarbon database. Paleoanthropology.

[95] Bronk Ramsey C (2009). Bayesian analysis of radiocarbon dates. Radiocarbon.

[96] Reimer PJ (2013). IntCal13 and Marine13 radiocarbon age calibration curves 0–50,000 years cal BP. Radiocarbon.

[97] Serjeantson D (1998). Birds: a seasonal resource. Environ. Archaeol..

[98] Serjeantson D (2009). Birds. Cambridge Manuals in Archaeology.

[99] Lima-Ribeiro MS (2015). EcoClimate: a database of climate data from multiple models for past, present, and future for macroecologists and biogeographers. Biodivers. Inform..

[100] Varela S, Lima-Ribeiro MS, Terribile LC (2015). A short guide to the climatic variables of the last glacial maximum for biogeographers. PLoS ONE.

[101] Elith J (2006). Novel methods improve prediction of species’ distributions from occurrence data. Ecography.

[102] Elith J, Leathwick JR, Hastie T (2008). A working guide to boosted regression trees. J. Anim. Ecol..

[103] Leathwick JR, Elith J, Francis MP, Hastie T, Taylor P (2006). Variation in demersal fish species richness in the oceans surrounding New Zealand: an analysis using boosted regression trees. Mar. Ecol. Prog. Ser..

[104] Leathwick JR, Elith J, Chadderton WL, Rowe D, Hastie T (2008). Dispersal, disturbance and the contrasting biogeographies of New Zealand’s diadromous and non-diadromous fish species. J. Biogeogr..

[105] Therneau, T. & Atkinson, B. *Rpart: Recursive Partitioning and Regression Trees. R package version 4.1-15*. https://CRAN.R-project.org/package=rpart (2019).

[106] Kuhn, M. *Caret: Classification and Regression Training. R package version 6.0-88*. https://CRAN.R-project.org/package=caret (2021).

