# Supplementary material for: Birds adapted to cold conditions show greater changes in range size related to past climatic oscillations than temperate birds
Source: Sci Rep. 2022 Jun 25;12:10813. doi: 10.1038/s41598-022-14972-7 (PMC9233688; doi:10.1038/s41598-022-14972-7)
Supplement: Supplementary file 1 — Supplementary Information. [file 41598_2022_14972_MOESM1_ESM.pdf]

Birds adapted to cold conditions show greater changes in range size related to past climatic oscillations than temperate birds

Lisa Carrera*[1], Marco Pavia[2], Sara Varela[3]

[1]Dipartimento di Scienze Biologiche, Geologiche e Ambientali, University of Bologna, Via Zamboni 67, 40126 Bologna, Italy

[2]Dipartimento di Scienze della Terra, Museo di Geologia e Paleontologia, University of Torino, Via Valperga Caluso 35, 10125 Torino, Italy

[3]Departamento de Ecoloxía e Bioloxía Animal, Universidade de Vigo, MAPAS Lab, 36310 Vigo, Spain

*Corresponding author

Supplementary Data S1 - Outline of the ecology of the six species investigated

P. graculus, a polytypic species, is a sedentary gregarious corvid that lives in high-altitude mountain areas of the Palearctic mid-latitudes, from the Pyrenees to the Himalaya. It inhabits pastures with cliffs and rocky ravines, descending below the treeline only in winter. Nests on ledges or shelfs near the roof of a cave, in rock crevices or on cliff faces[1, 2]. This species is known in the Palearctic since the Early Pleistocene and becomes very common in the European Late Pleistocene localities[3,4].

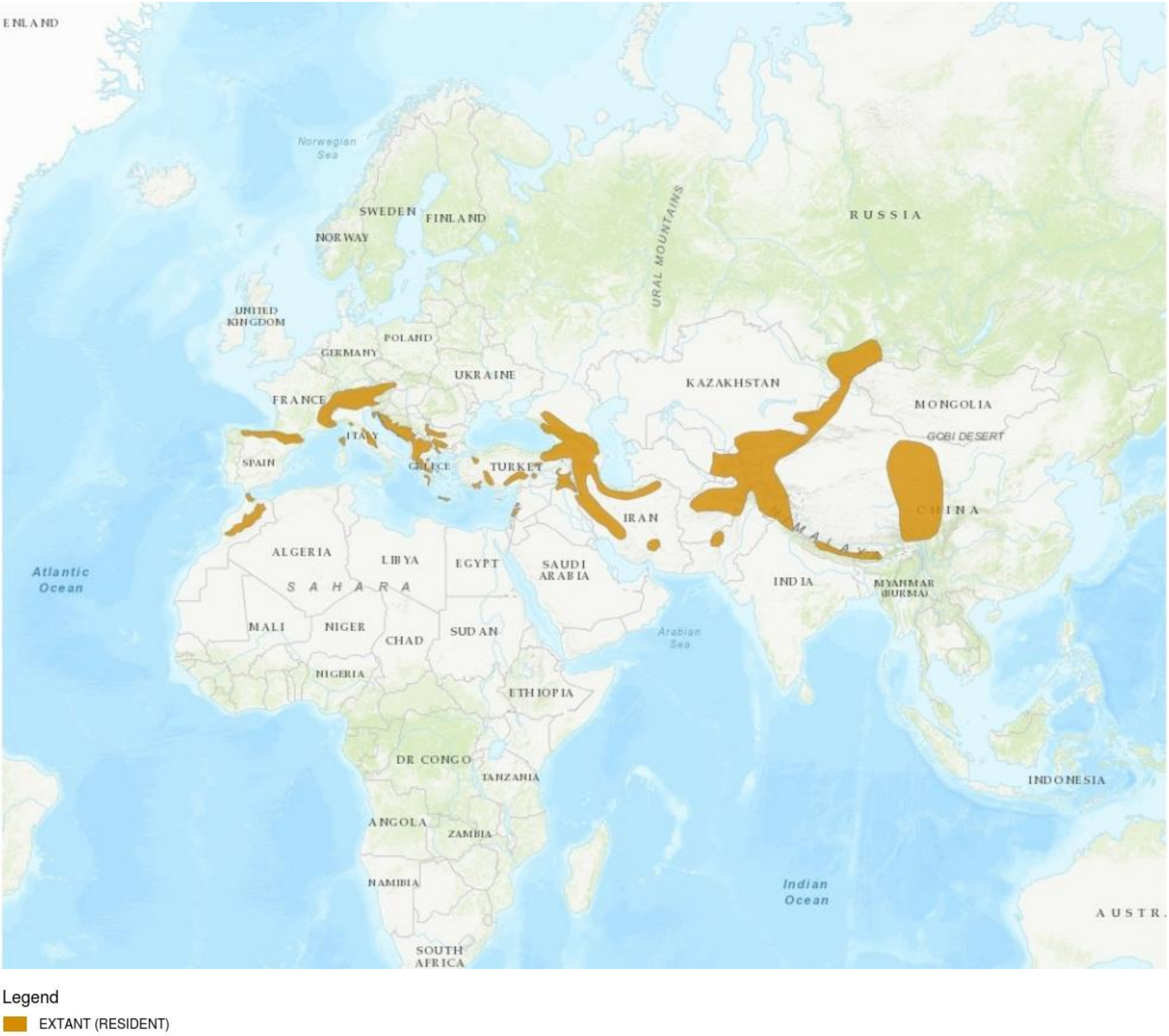

P. graculus present-day distribution. The map was downloaded and modified from the IUCN website (*Pyrrhocorax graculus* Species assessment)[1].

B. scandiacus is a monotypic large owl that breeds in the Arctic regions of Eurasia and America, in the open tundra with sparse low vegetation, in coastal fields or open moorland. The nest is a shallow scrape on ground. The species is mostly migratory and nomadic, even if some individuals remain in the breeding areas all year round. In winter, birds move southwards in Northern Eurasia and North America, due to abundance of prey species (mainly lemmings and small voles), that causes the so called 'snowy owl irruptions'. The species is listed as vulnerable as it is undergoing rapid population decline[2, 5]. *B. scandiacus* is reported in the Palearctic fossil record since the Early Pleistocene[3,4].

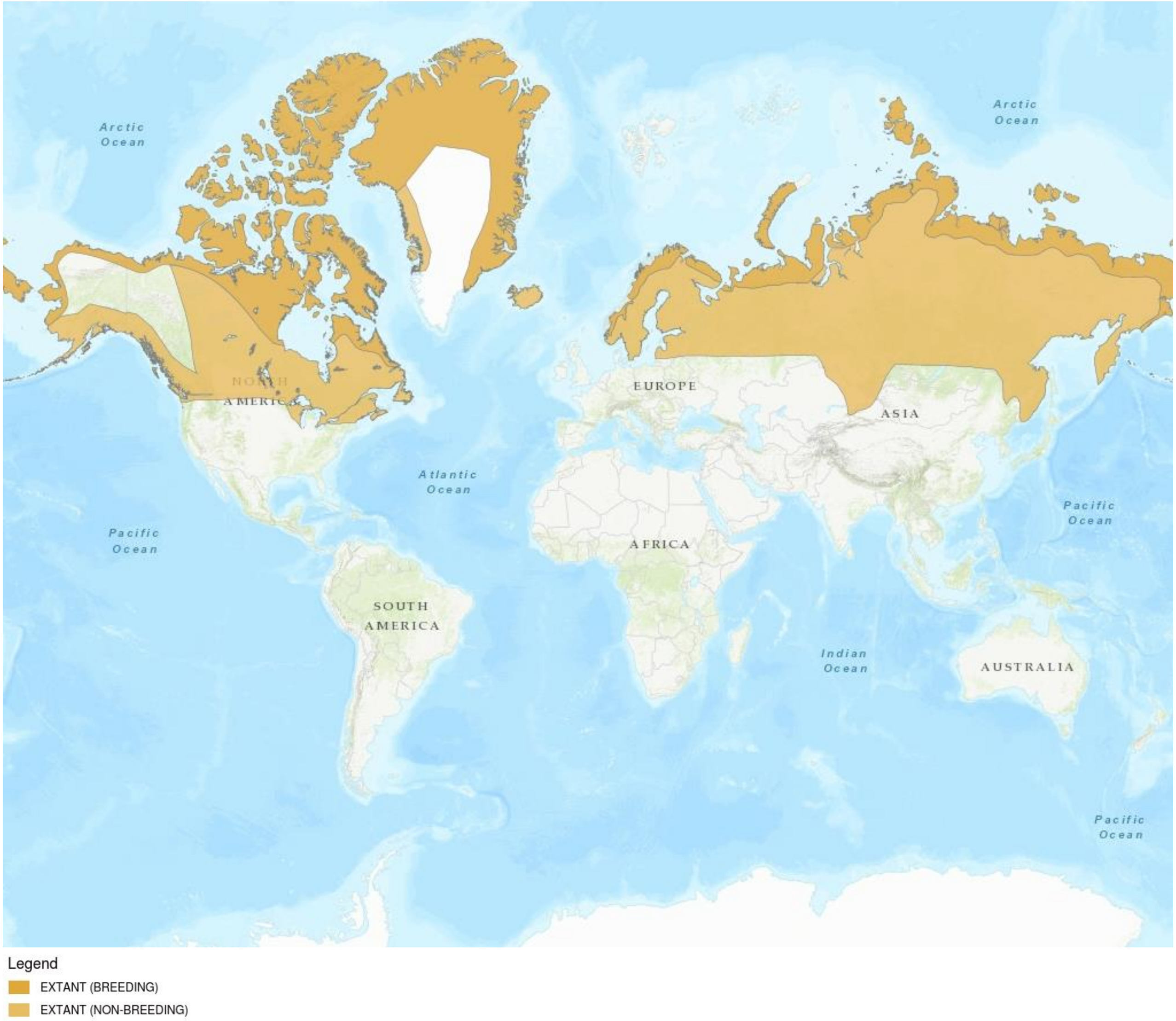

B. scandiacus present-day distribution. The map was downloaded and modified from the IUCN website (*Bubo scandiacus* Species assessment)[5].

A. noctua is a polytypic common small sedentary owl which is spread in a variety of semi-open habitats, from parklands to semi-desert regions, spanning from boreal to tropical areas of Eurasia and Northern Africa but preferring in general warm arid areas. It nests in cavities[2, 6]. This species is known in the fossil record since the Early Pleistocene[3,4].

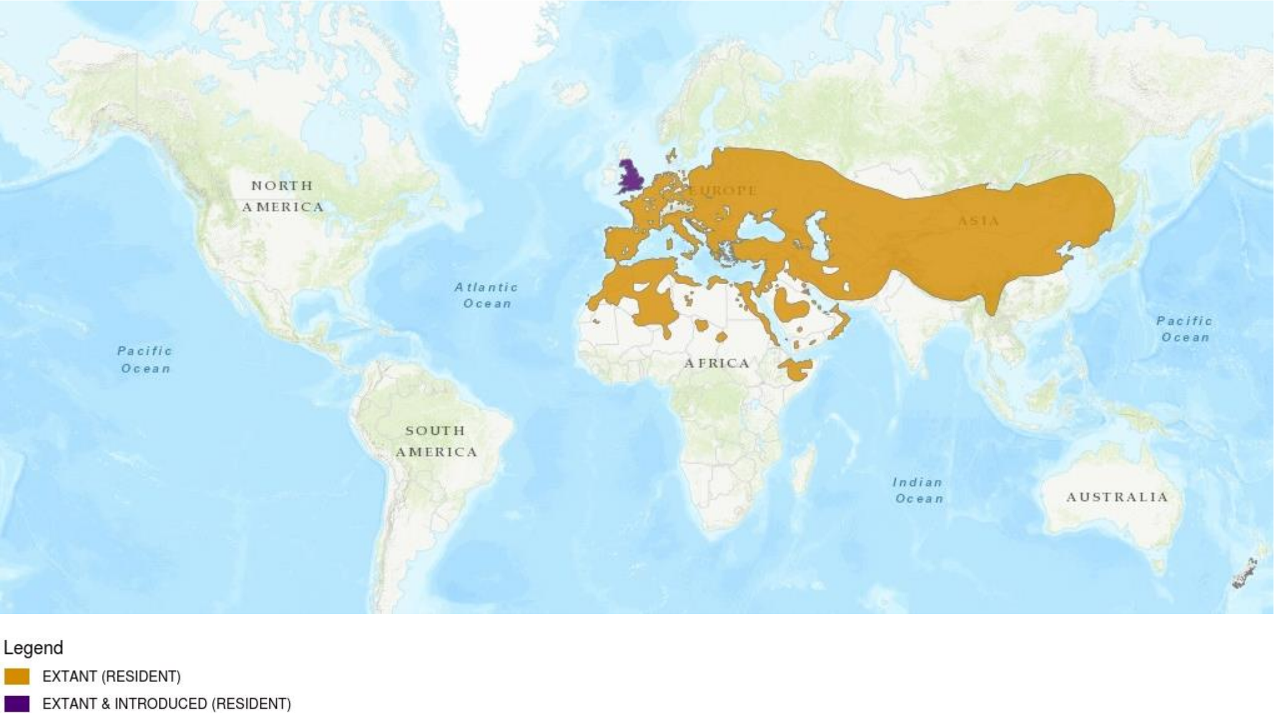

A. noctua present-day distribution. The map was downloaded and modified from the IUCN website (*Athene noctua* Species assessment)[6].

P. *perdix* is a polytypic medium-sized sedentary Galliformes which occurs throughout much of the Western Palearctic. This species has suffered from massive declines during the XXth century owing to habitat loss and degradation caused by agricultural intensification. It is found in grasslands with some dense shrubby patches and nests in a shallow depression at the base of a hedge or other thick vegetation[2, 7]. *P. perdix* is reported in the Palearctic fossil record since the Early Pleistocene, being more abundant in the Late Pleistocene[3,4].

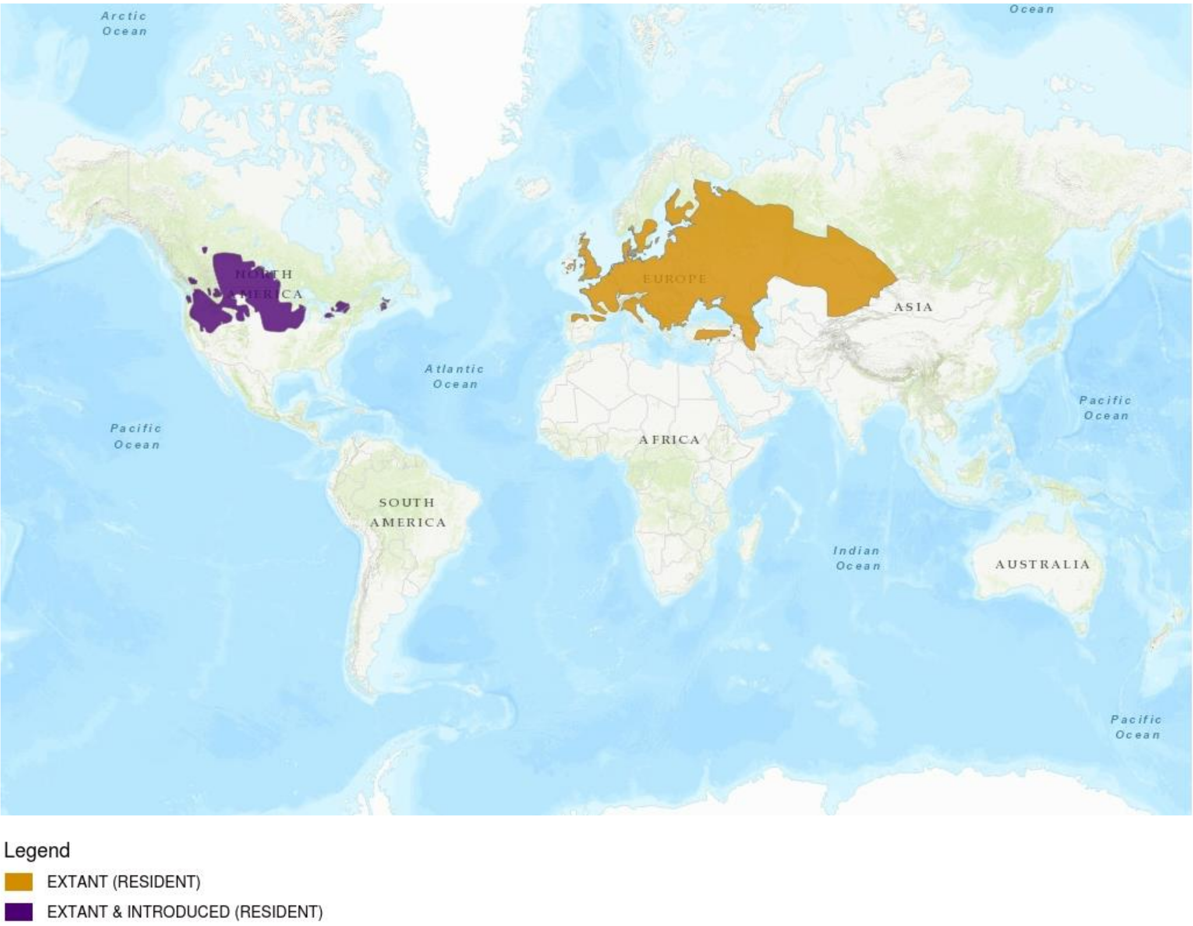

P. *perdix* present-day distribution. The map was downloaded and modified from the IUCN website (*Perdix perdix* Species assessment)[7].

C. crex, a monotypic medium-sized rail, is a full long-distance migrant which breeds in Europe and central Asia, in open or semi-open environments, such as meadows with tall grass. The nest is on the ground, in dense vegetation. This species winters in eastern sub-Saharan Africa, where prefers dry grasslands and savannas[2, 8]. This species is known since the Early Pleistocene, becoming more abundant in the Late Pleistocene[3,4].

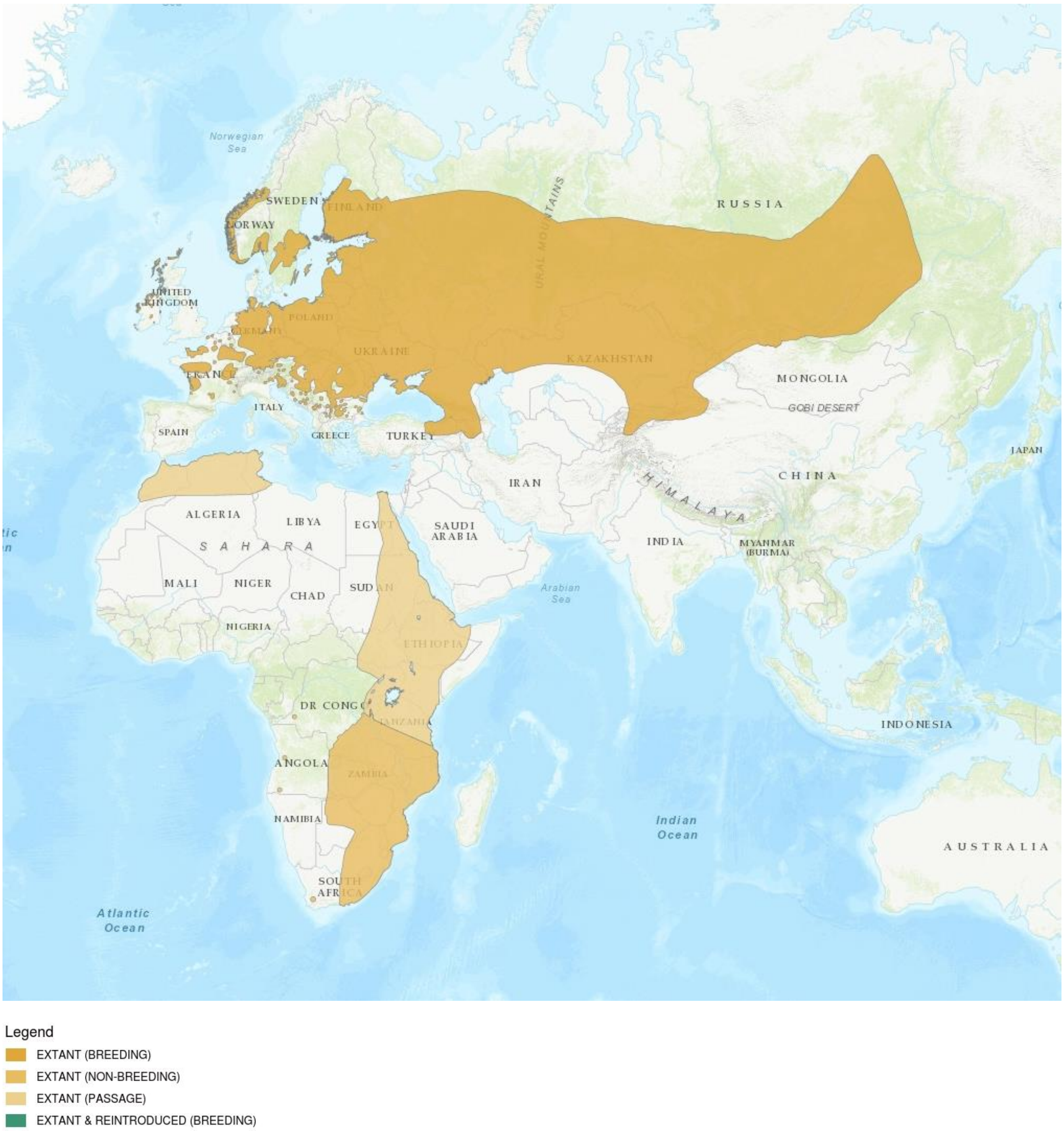

C. crex present-day distribution. The map was downloaded and modified from the IUCN website (*Crex crex* Species assessment)[8].

C. coturnix is a polytypic small Galliformes that breeds in the open grasslands of Eurasia. The nest is a scrape in herbaceous vegetation on the ground. It is a full long-distance migrant and spends the winters in sub-Saharan Africa, mainly in the Sahel zone[2, 9]. *C. coturnix* is known in the Palearctic fossil record since the Early Pleistocene and becomes more common in the Late Pleistocene[3,4].

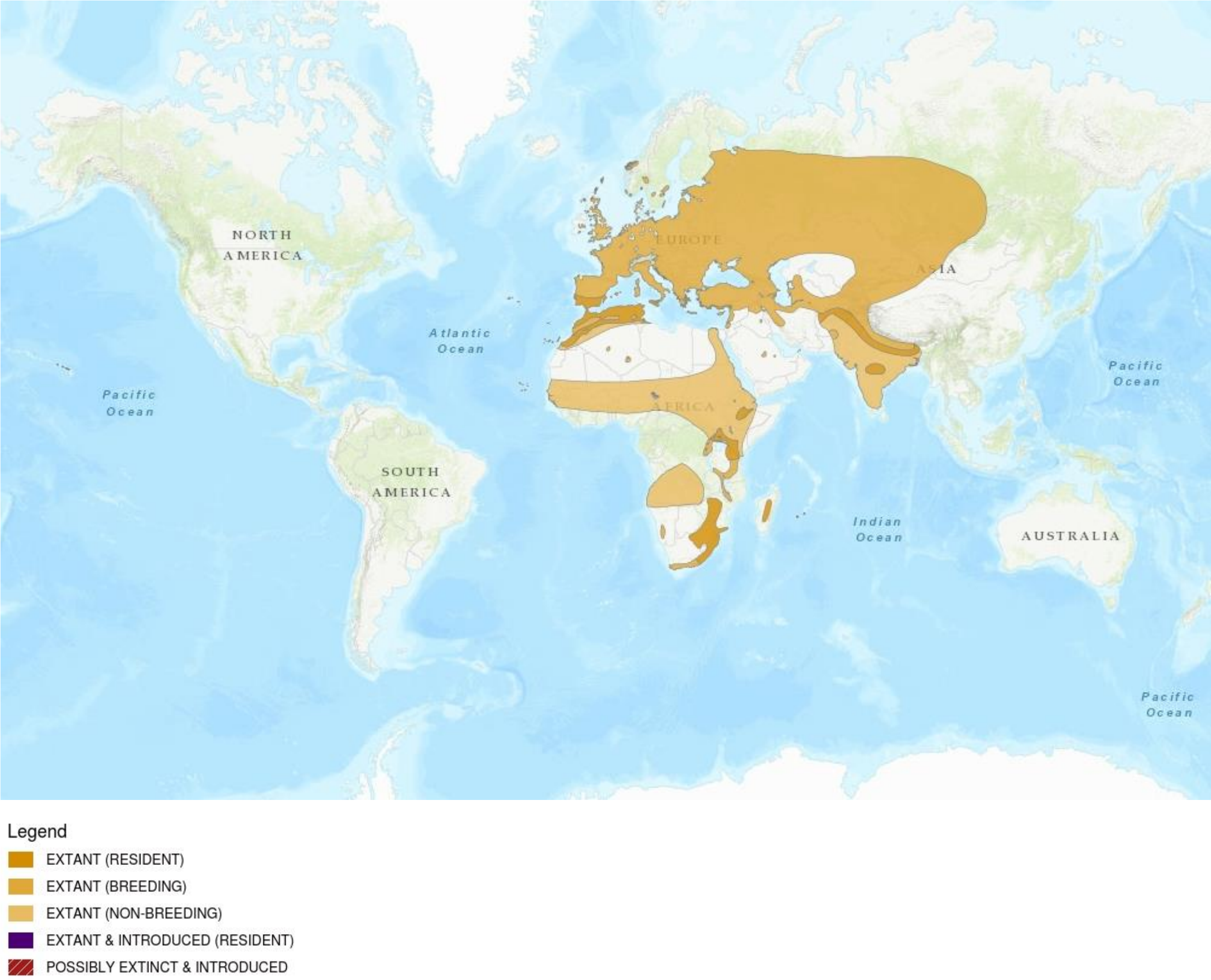

C. coturnix present-day distribution. The map was downloaded and modified from the IUCN website (*Coturnix coturnix* Species assessment)[9].

Supplementary Data S2 - Variable importance (relative influence of the five more important variables) and evaluation values of each model (CCSM, CNRM, FGOALS, GISS, IPSL, MIROC, MPI, MRI) for each species

Pyrrhocorax graculus **- CCSM**

Var. importance: bio.8 18.3800831, bio.3 16.0713235, bio.2 8.3971183, bio.18 7.5067773, bio.9 7.1362126

Evaluation train
n presences : 237
n absences : 244
AUC : 1
cor : 0.9921315
max TPR+TNR at : 0.6151186

Evaluation test
n presences : 63
n absences : 56
AUC : 0.973356
cor : 0.8780461
max TPR+TNR at : 0.5027866

Pyrrhocorax graculus **- CNRM**

Var. importance: bio.3 22.9945136, bio.2 16.0200376, bio.8 10.9674794, bio.19 7.9610327, bio.13 6.6381431

Evaluation train
n presences : 239
n absences : 242
AUC : 1
cor : 0.9960988
max TPR+TNR at : 0.7164497

Evaluation test
n presences : 61
n absences : 58

AUC : 0.9946297
cor : 0.9425577
max TPR+TNR at : 0.6161275

***Pyrrhocorax graculus* - FGOALS**

Var. importance: bio.3 28.37778402, bio.8 15.79259811, bio.19 12.74193031, bio.11 8.84796531, bio.12 6.80968332

Evaluation train
n presences : 240
n absences : 240
AUC : 1
cor : 0.9934999
max TPR+TNR at : 0.7328617

Evaluation test
n presences : 60
n absences : 60
AUC : 0.9752778
cor : 0.9166899
max TPR+TNR at : 0.6807326

***Pyrrhocorax graculus* - GISS**

Var. importance: bio.3 26.4024333, bio.8 11.4122872, bio.4 9.6939950, bio.19 8.9409073, bio.2 7.3981664

Evaluation train
n presences : 246
n absences : 236
AUC : 0.9999139
cor : 0.9845505
max TPR+TNR at : 0.6835134

Evaluation test
n presences : 54
n absences : 64

AUC   : 0.9748264
cor   : 0.8819202
max TPR+TNR at : 0.631979

Pyrrhocorax graculus **- IPSL**

Var. importance: bio.3 27.3497044, bio.19 12.8601738, bio.5 10.7005723, bio.4 8.6736239, bio.14 8.0101202

Evaluation train
class   : ModelEvaluation
n presences   : 245
n absences   : 235
AUC   : 1
cor   : 0.9987745
max TPR+TNR at : 0.78706

Evaluation test
n presences   : 55
n absences   : 65
AUC   : 0.9876923
cor   : 0.9206223
max TPR+TNR at : 0.4379987

Pyrrhocorax graculus **- MIROC**

Var. importance: bio.3 13.9051970, bio.8 11.4598980, bio.19 9.2772188, bio.13 9.0855225, bio.2 8.9160167

Evaluation train
n presences   : 244
n absences   : 236
AUC   : 1
cor   : 0.9916561
max TPR+TNR at : 0.686271

Evaluation test

n presences   : 56

n absences   : 64

AUC   : 0.9986049

cor   : 0.9600088

max TPR+TNR at : 0.6693516

***Pyrrhocorax graculus* - MPI**

Var. importance: bio.3 40.2250167, bio.8 12.0572192, bio.2 9.6545252, bio.6 6.1232959, bio.19 6.0636037

Evaluation train

n presences   : 240

n absences   : 240

AUC   : 0.9999826

cor   : 0.9877139

max TPR+TNR at : 0.4446355

Evaluation test

n presences   : 60

n absences   : 60

AUC   : 0.9725

cor   : 0.8857112

max TPR+TNR at : 0.2923693

***Pyrrhocorax graculus* - MRI**

Var. importance: bio.4 17.0415457, bio.3 14.3266886, bio.13 9.4924036, bio.8 9.1918337, bio.15 6.8687489

Evaluation train

n presences   : 255

n absences   : 225

AUC   : 0.9932026

cor   : 0.9349929

max TPR+TNR at : 0.6041073

Evaluation test

n presences : 45

n absences : 75

AUC : 0.938963

cor : 0.7652218

max TPR+TNR at : 0.5930847

***Bubo scandiacus*, wintering range - CCSM**

Var. importance: bio.7 27.2239878, bio.1 15.5909058, bio.14 9.4530419, bio.15 7.3569528, bio.12 7.3277208

Evaluation train

n presences : 240

n absences : 240

AUC : 1

cor : 0.9991487

max TPR+TNR at : 0.8312412

Evaluation test

n presences : 60

n absences : 60

AUC : 0.9963889

cor : 0.9521732

max TPR+TNR at : 0.8562762

***Bubo scandiacus*, wintering range - CNRM**

Var. importance: bio.3 16.7455687, bio.2 15.8073993, bio.8 14.4689629, bio.18 11.2705948, bio.4 7.4172494

Evaluation train

n presences : 242

n absences : 238

AUC : 1

cor : 0.9976284

max TPR+TNR at : 0.7211729

Evaluation test
n presences : 58
n absences : 62
AUC : 0.9799778
cor : 0.9063431
max TPR+TNR at : 0.5861419

***Bubo scandiacus*, wintering range - FGOALS**

Var. importance: bio.1 21.07941739, bio.2 12.44932914, bio.5 11.57684057, bio.4 10.92289095, bio.10 9.78132705

Evaluation train
n presences : 237
n absences : 243
AUC : 1
cor : 0.9986841
max TPR+TNR at : 0.8216523

Evaluation test
n presences : 63
n absences : 57
AUC : 0.9885826
cor : 0.93763
max TPR+TNR at : 0.389507

***Bubo scandiacus*, wintering range – GISS**

Var. importance: bio.4 39.0772485, bio.12 16.3543840, bio.3 8.5482351, bio.1 7.1132717, bio.13 4.8158901

Evaluation train
n presences : 230
n absences : 250
AUC : 1
cor : 0.9986837

max TPR+TNR at : 0.7920148

Evaluation test
n presences : 70
n absences : 50
AUC : 0.9945714
cor : 0.9445038
max TPR+TNR at : 0.468201

***Bubo scandiacus*, wintering range - IPSL**

Var. importance: bio.4 21.3357454, bio.3 14.5815777, bio.12 10.3697115, bio.8 9.9929003, bio.15 9.3718031

Evaluation train
n presences : 239
n absences : 241
AUC : 1
cor : 0.9988687
max TPR+TNR at : 0.8172766

Evaluation test
n presences : 61
n absences : 59
AUC : 0.9886079
cor : 0.9344117
max TPR+TNR at : 0.2361584

***Bubo scandiacus*, wintering range - MIROC**

Var. importance: bio.3 24.9953274, bio.1 18.2076517, bio.15 12.9824335, bio.4 6.0116626, bio.7 5.9562372

Evaluation train
n presences : 240
n absences : 240
AUC : 1

cor : 0.9985086

max TPR+TNR at : 0.7472241

Evaluation test

n presences : 60

n absences : 60

AUC : 0.9977778

cor : 0.9587645

max TPR+TNR at : 0.3506854

***Bubo scandiacus*, wintering range – MPI**

Var. importance: bio.1 26.4607327, bio.18 12.4462073, bio.2 12.1054681, bio.4 7.5533832, bio.12 6.2563012

Evaluation train

n presences : 240

n absences : 240

AUC : 1

cor : 0.9982287

max TPR+TNR at : 0.7281611

Evaluation test

n presences : 60

n absences : 60

AUC : 0.9966667

cor : 0.9590535

max TPR+TNR at : 0.3749381

***Bubo scandiacus*, wintering range – MRI**

Var. importance: bio.1 21.6283396, bio.4 18.9615116, bio.2 8.7262568, bio.12 8.4894958, bio.14 6.7380018

Evaluation train

n presences : 245

n absences : 235

AUC : 1
cor : 0.997647
max TPR+TNR at : 0.7425055

Evaluation test
n presences : 55
n absences : 65
AUC : 0.9963636
cor : 0.9506461
max TPR+TNR at : 0.7300653

***Bubo scandiacus*, breeding range - CCSM**

Var. importance: bio.10 25.5475642, bio.5 21.6367823, bio.2 13.4077419, bio.3 11.5905906, bio.18 7.5116805

Evaluation train
n presences : 230
n absences : 250
AUC : 1
cor : 0.9957085
max TPR+TNR at : 0.5962335

Evaluation test
n presences : 70
n absences : 50
AUC : 0.9928571
cor : 0.9434722
max TPR+TNR at : 0.4396043

***Bubo scandiacus*, breeding range - CNRM**

Var. importance: bio.2 38.86506839, bio.10 12.13846465, bio.18 11.54050024, bio.3 10.50409802, bio.8 10.02260932

Evaluation train
n presences : 241

n absences : 239

AUC : 1

cor : 0.9962053

max TPR+TNR at : 0.7298184

Evaluation test

n presences : 59

n absences : 61

AUC : 0.9936093

cor : 0.9630463

max TPR+TNR at : 0.6134392

***Bubo scandiacus*, breeding range - FGOALS**

Var. importance: bio.2 63.023398182, bio.18 12.794795556, bio.3 5.772470297, bio.7 5.323328034, bio.5 3.428982334

Evaluation train

n presences : 242

n absences : 238

AUC : 1

cor : 0.9974436

max TPR+TNR at : 0.7373109

Evaluation test

n presences : 58

n absences : 62

AUC : 0.996941

cor : 0.9523647

max TPR+TNR at : 0.6373854

***Bubo scandiacus*, breeding range - GISS**

Var. importance: bio.8 35.13853357, bio.3 14.67880610, bio.10 14.28602553, bio.18 13.13751125, bio.9 5.57046846

Evaluation train

n presences : 242

n absences : 239

AUC : 0.9999308

cor : 0.9865104

max TPR+TNR at : 0.4385703

Evaluation test

n presences : 58

n absences : 61

AUC : 1

cor : 0.9753737

max TPR+TNR at : 0.4011146

***Bubo scandiacus*, breeding range - IPSL**

Var. importance: bio.18 41.52832071, bio.5 17.73191658, bio.10 16.49978445, bio.3 9.11739245, bio.16 3.17442494

Evaluation train

n presences : 235

n absences : 245

AUC : 0.9999826

cor : 0.9941468

max TPR+TNR at : 0.3883646

Evaluation test

n presences : 65

n absences : 55

AUC : 0.9888112

cor : 0.9730529

max TPR+TNR at : 0.4870946

***Bubo scandiacus*, breeding range - MIROC**

Var. importance: bio.2 32.4750836, bio.18 26.8869046, bio.5 13.0079504, bio.3 7.7053595, bio.4 4.3097379

Evaluation train

n presences : 241
n absences : 239
AUC : 1
cor : 0.9942988
max TPR+TNR at : 0.6493983

Evaluation test
n presences : 59
n absences : 61
AUC : 0.9966657
cor : 0.9415295
max TPR+TNR at : 0.3168652

***Bubo scandiacus*, breeding range - MPI**

Var. importance: bio.18 31.87432986, bio.10 19.40992072, bio.8 14.39910127, bio.3 7.68145144, bio.5 6.31720107

Evaluation train
n presences : 242
n absences : 238
AUC : 1
cor : 0.9955193
max TPR+TNR at : 0.6977875

Evaluation test
n presences : 58
n absences : 62
AUC : 0.9986096
cor : 0.9691946
max TPR+TNR at : 0.5012148

***Bubo scandiacus*, breeding range - MRI**

Var. importance: bio.2 29.69420535, bio.18 19.04505073, bio.3 12.70456817, bio.10 8.98578139, bio.6 7.32160908

Evaluation train
n presences : 241
n absences : 239
AUC : 1
cor : 0.9930614
max TPR+TNR at : 0.4734608

Evaluation test
n presences : 59
n absences : 61
AUC : 0.9944429
cor : 0.944481
max TPR+TNR at : 0.8194517

***Crex crex*, wintering range - CCSM**

Var. importance: bio.4 30.282804785, bio.2 17.200564045, bio.3 11.807693951, bio.12 9.216402669, bio.18 5.777278533

Evaluation train
n presences : 238
n absences : 243
AUC : 1
cor : 0.9940242
max TPR+TNR at : 0.3054828

Evaluation test
n presences : 62
n absences : 57
AUC : 0.999717
cor : 0.9740611
max TPR+TNR at : 0.699541

***Crex crex*, wintering range - CNRM**

Var. importance: bio.4 46.100070457, bio.7 17.573725700, bio.13 14.510444670, bio.12 4.131934302, bio.1 3.413672391

Evaluation train
n presences : 234
n absences : 247
AUC : 1
cor : 0.9995034
max TPR+TNR at : 0.8145048

Evaluation test
n presences : 66
n absences : 53
AUC : 0.9874214
cor : 0.9336682
max TPR+TNR at : 0.6544742

***Crex crex*, wintering range - FGOALS**

Var. importance: bio.2 23.97015456, bio.3 19.32741040, bio.15 18.81200863, bio.19 10.27772809, bio.14 7.84980450

Evaluation train
n presences : 235
n absences : 246
AUC : 1
cor : 0.9983678
max TPR+TNR at : 0.6886163

Evaluation test
n presences : 65
n absences : 54
AUC : 0.9985755
cor : 0.9359414
max TPR+TNR at : 0.1149004

***Crex crex*, wintering range – GISS**

Var. importance: bio.4 17.25297635, bio.2 16.46719569, bio.9 14.59587665, bio.3 13.29858952, bio.18 7.62853476

Evaluation train
n presences : 241
n absences : 240
AUC : 1
cor : 0.9991592
max TPR+TNR at : 0.8454622

Evaluation test
n presences : 59
n absences : 60
AUC : 0.9960452
cor : 0.9693653
max TPR+TNR at : 0.6386525

***Crex crex*, wintering range - IPSL**

Var. importance: bio.3 20.46562367, bio.4 17.76917680, bio.14 15.34935657, bio.12 10.92635502, bio.15 7.43944547

Evaluation train
n presences : 246
n absences : 235
AUC : 1
cor : 0.9998264
max TPR+TNR at : 0.8678773

Evaluation test
n presences : 54
n absences : 65
AUC : 1
cor : 0.9850954
max TPR+TNR at : 0.09515892

***Crex crex*, wintering range – MIROC**

Var. importance: bio.4 55.26914083, bio.2 23.36702002, bio.3 10.06728435, bio.8 2.76942724, bio.15 2.58282018

Evaluation train
n presences   : 236
n absences   : 245
AUC   : 1
cor   : 0.9994265
max TPR+TNR at : 0.8540138

Evaluation test
n presences   : 64
n absences   : 55
AUC   : 0.9875
cor   : 0.9327763
max TPR+TNR at : 0.8809668

***Crex crex*, wintering range - MPI**

Var. importance: bio.4 45.943304712, bio.2 12.581368900, bio.6 7.533858253, bio.12 4.961918245, bio.7 4.766432485

Evaluation train
n presences   : 234
n absences   : 247
AUC   : 1
cor   : 0.9987048
max TPR+TNR at : 0.7589877

Evaluation test
n presences   : 66
n absences   : 53
AUC   : 1
cor   : 0.9771309
max TPR+TNR at : 0.3681444

***Crex crex*, wintering range - MRI**

Var. importance: bio.4 29.57187182, bio.2 27.35057617, bio.7 9.25284430, bio.5 6.21539381, bio.9 5.41813102

Evaluation train
n presences   : 237
n absences   : 243
AUC   : 1
cor   : 0.9991929
max TPR+TNR at : 0.846166

Evaluation test
n presences   : 63
n absences   : 57
AUC   : 0.9991646
cor   : 0.9742106
max TPR+TNR at : 0.5849

***Crex crex*, breeding range - CCSM**

Var. importance: bio.14 20.9035783, bio.17 18.6809529, bio.15 12.7963315, bio.10 12.0868336, bio.5 5.2841321

Evaluation train
n presences   : 243
n absences   : 238
AUC   : 0.9975101
cor   : 0.9705384
max TPR+TNR at : 0.4707432

Evaluation test
n presences   : 57
n absences   : 62
AUC   : 0.9949066
cor   : 0.9324419
max TPR+TNR at : 0.6328612

***Crex crex*, breeding range - CNRM**

Var. importance: bio.14 22.32631002, bio.3 21.98682569, bio.15 21.46837243, bio.5 13.38097802, bio.17 4.24931944

Evaluation train
n presences : 238
n absences : 242
AUC : 0.9997569
cor : 0.990436
max TPR+TNR at : 0.5261475

Evaluation test
n presences : 62
n absences : 58
AUC : 0.9849833
cor : 0.9171823
max TPR+TNR at : 0.5777403

***Crex crex*, breeding range - FGOALS**

Var. importance: bio.15 44.94148718, bio.3 16.28276996, bio.7 9.85999415, bio.5 6.16598337, bio.10 5.63304800

Evaluation train
n presences : 240
n absences : 240
AUC : 1
cor : 0.9963634
max TPR+TNR at : 0.5978673

Evaluation test
n presences : 60
n absences : 60
AUC : 0.9961111
cor : 0.9423702

max TPR+TNR at : 0.2245815

***Crex crex*, breeding range - GISS**

Var. importance: bio.14 30.5778367, bio.3 18.5401210, bio.15 12.1416935, bio.5 6.0467565, bio.2 5.2791969

Evaluation train
n presences : 241
n absences : 239
AUC : 1
cor : 0.998038
max TPR+TNR at : 0.7249346

Evaluation test
n presences : 59
n absences : 61
AUC : 0.9872187
cor : 0.8870871
max TPR+TNR at : 0.4323176

***Crex crex*, breeding range - IPSL**

Var. importance: bio.14 25.1566165, bio.15 13.5453563, bio.10 13.1348398, bio.3 11.3211041, bio.17 9.1949693

Evaluation train
n presences : 234
n absences : 246
AUC : 0.9997915
cor : 0.9858515
max TPR+TNR at : 0.4932286

Evaluation test
n presences : 66
n absences : 54
AUC : 0.9963524

cor : 0.952546

max TPR+TNR at : 0.7546186

***Crex crex*, breeding range - MIROC**

Var. importance: bio.14 31.5639239, bio.15 12.8959047, bio.10 12.2342087, bio.3 11.2694059, bio.2 11.0070826

Evaluation train

n presences : 242

n absences : 239

AUC : 0.9999481

cor : 0.9888414

max TPR+TNR at : 0.3116323

Evaluation test

n presences : 58

n absences : 61

AUC : 0.9771057

cor : 0.8814432

max TPR+TNR at : 0.7827192

***Crex crex*, breeding range - MPI**

Var. importance: bio.17 21.2818417, bio.15 21.2607932, bio.3 16.3947449, bio.5 12.1971519, bio.9 4.9545459

Evaluation train

n presences : 245

n absences : 235

AUC : 0.9988363

cor : 0.9767157

max TPR+TNR at : 0.4597665

Evaluation test

n presences : 55

n absences : 65

AUC : 0.993007
cor : 0.9463434
max TPR+TNR at : 0.3621413

***Crex crex*, breeding range – MRI**

Var. importance: bio.14 36.7130578, bio.15 16.7919640, bio.10 16.1923290, bio.3 6.7294319, bio.17 4.3306131

Evaluation train
n presences : 242
n absences : 238
AUC : 1
cor : 0.9932859
max TPR+TNR at : 0.2813261

Evaluation test
n presences : 58
n absences : 62
AUC : 0.9655172
cor : 0.8888126
max TPR+TNR at : 0.8022112

***Coturnix coturnix*, wintering range - CCSM**

Var. importance: bio.4 17.9623448, bio.5 11.9151820, bio.8 10.4937027, bio.10 8.8285430, bio.6 7.2447972

Evaluation train
n presences : 242
n absences : 238
AUC : 0.9975693
cor : 0.9657115
max TPR+TNR at : 0.4988285

Evaluation test
n presences : 58

n absences : 62
AUC : 0.9746941
cor : 0.8746813
max TPR+TNR at : 0.3329008

***Coturnix coturnix*, wintering range - CNRM**

Var. importance: bio.4 21.7372095, bio.7 9.9724024, bio.5 8.4476829, bio.16 7.3428954, bio.10 6.8990452

Evaluation train
n presences : 236
n absences : 244
AUC : 0.9976035
cor : 0.9556678
max TPR+TNR at : 0.419839

Evaluation test
n presences : 64

n absences : 56
AUC : 0.9933036
cor : 0.9206238
max TPR+TNR at : 0.6936789

***Coturnix coturnix*, wintering range - FGOALS**

Var. importance: bio.7 16.1020922, bio.1 10.5326047, bio.12 10.0178285, bio.18 8.5837885, bio.9 8.2434392

Evaluation train
n presences : 243
n absences : 237
AUC : 0.9998958
cor : 0.983663
max TPR+TNR at : 0.5491697

Evaluation test

n presences : 57

n absences : 63

AUC : 0.9922027

cor : 0.9242059

max TPR+TNR at : 0.5933

***Coturnix coturnix*, wintering range - GISS**

Var. importance: bio.3 18.1918222, bio.1 11.9648465, bio.7 8.5846512, bio.4 6.8984376, bio.2 5.9054104

Evaluation train

n presences : 246

n absences : 234

AUC : 0.9999131

cor : 0.9819429

max TPR+TNR at : 0.4765673

Evaluation test

n presences : 54

n absences : 66

AUC : 0.9329405

cor : 0.7425909

max TPR+TNR at : 0.6442904

***Coturnix coturnix*, wintering range - IPSL**

Var. importance: bio.7 29.245184, bio.12 12.148565, bio.17 10.662309, bio.6 7.989266, bio.5 6.187066

Evaluation train

n presences : 237

n absences : 243

AUC : 1

cor : 0.9936169

max TPR+TNR at : 0.650407

Evaluation test

n presences   : 63
n absences   : 57
AUC   : 0.9944305
cor   : 0.9523136
max TPR+TNR at : 0.48306

***Coturnix coturnix*, wintering range - MIROC**

Var. importance: bio.6 14.1203613, bio.12 12.9834998, bio.4 11.8273751, bio.8 11.3047986, bio.5 8.9737631

Evaluation train
n presences   : 248
n absences   : 232
AUC   : 1
cor   : 0.9901551
max TPR+TNR at : 0.5562421

Evaluation test
n presences   : 52
n absences   : 68
AUC   : 0.9889706
cor   : 0.9044362
max TPR+TNR at : 0.4181047

***Coturnix coturnix*, wintering range - MPI**

Var. importance: bio.8 20.498409, bio.4 16.256101, bio.12 10.020766, bio.2 7.398379, bio.19 4.763113

Evaluation train
n presences   : 238
n absences   : 242
AUC   : 1
cor   : 0.9848642
max TPR+TNR at : 0.5068185

Evaluation test

n presences : 62
n absences : 58
AUC : 0.9858176
cor : 0.9163195
max TPR+TNR at : 0.4543837

***Coturnix coturnix*, wintering range – MRI**

Var. importance: bio.4 21.0393872, bio.1 16.8986830, bio.18 7.8305022, bio.7 7.8164008, bio.19 6.3770153

Evaluation train
n presences : 237
n absences : 243
AUC : 0.9990624
cor : 0.969939
max TPR+TNR at : 0.4817052

Evaluation test
n presences : 63
n absences : 57
AUC : 0.9651908
cor : 0.8370769
max TPR+TNR at : 0.6013944

***Coturnix coturnix*, breeding range - CCSM**

Var. importance: bio.19 29.2223697, bio.10 13.8134451, bio.1 8.9074008, bio.17 7.3405455, bio.8 6.6596123

Evaluation train
n presences : 236
n absences : 244
AUC : 0.9997916
cor : 0.9788055
max TPR+TNR at : 0.431902

Evaluation test

n presences : 64

n absences : 56

AUC : 0.96875

cor : 0.8673039

max TPR+TNR at : 0.8677223

***Coturnix coturnix*, breeding range - CNRM**

Var. importance: bio.17 17.9656688, bio.14 16.4324267, bio.15 14.6303822, bio.5 9.1382797, bio.2 6.7243966

Evaluation train

n presences : 237

n absences : 243

AUC : 0.999236

cor : 0.9698913

max TPR+TNR at : 0.4222124

Evaluation test

n presences : 63

n absences : 57

AUC : 0.9908104

cor : 0.9301162

max TPR+TNR at : 0.4307516

***Coturnix coturnix*, breeding range - FGOALS**

Var. importance: bio.15 27.4980444, bio.17 11.5847130, bio.10 8.7452647, bio.14 8.4438302, bio.5 7.6339922

Evaluation train

n presences : 238

n absences : 242

AUC : 1

cor : 0.9953713

max TPR+TNR at : 0.5848919

Evaluation test

n presences : 62

n absences : 58

AUC : 0.9769188

cor : 0.9233469

max TPR+TNR at : 0.8746123

***Coturnix coturnix*, breeding range - GISS**

Var. importance: bio.15 24.0487664, bio.17 12.8176999, bio.10 11.3415014, bio.5 9.7553471, bio.1 7.4547185

Evaluation train

n presences : 238

n absences : 242

AUC : 0.9995139

cor : 0.9744827

max TPR+TNR at : 0.4289757

Evaluation test

n presences : 62

n absences : 58

AUC : 0.958287

cor : 0.8606796

max TPR+TNR at : 0.7558889

***Coturnix coturnix*, breeding range - IPSL**

Var. importance: bio.19 23.0988953, bio.17 14.5998462, bio.5 11.7479505, bio.10 9.2589389, bio.15 7.6796715

Evaluation train

n presences : 242

n absences : 238

AUC : 1

cor : 0.989864

max TPR+TNR at : 0.421792

Evaluation test
n presences : 58
n absences : 62
AUC : 0.9938821
cor : 0.9319797
max TPR+TNR at : 0.1546534

***Coturnix coturnix*, breeding range - MIROC**

Var. importance: bio.19 19.5174500, bio.5 14.3639640, bio.17 12.1129567, bio.14 9.6495770, bio.3 7.0437288

Evaluation train
n presences : 236
n absences : 244
AUC : 1
cor : 0.9929026
max TPR+TNR at : 0.4988432

Evaluation test
n presences : 64
n absences : 56
AUC : 0.9662388
cor : 0.8421749
max TPR+TNR at : 0.5554222

***Coturnix coturnix*, breeding range - MPI**

Var. importance: bio.19 27.8303278, bio.15 16.0398969, bio.5 15.7456840, bio.8 6.3393267, bio.2 5.8255246

Evaluation train
n presences : 242
n absences : 238
AUC : 0.9999306

cor : 0.9834607

max TPR+TNR at : 0.5833983

Evaluation test

n presences : 58

n absences : 62

AUC : 0.987208

cor : 0.9233983

max TPR+TNR at : 0.4652753

***Coturnix coturnix*, breeding range - MRI**

Var. importance: bio.17 44.0342040, bio.10 10.6438399, bio.19 9.0906091, bio.3 7.6872797, bio.5 4.5419607

Evaluation train

n presences : 241

n absences : 239

AUC : 0.9993229

cor : 0.9721603

max TPR+TNR at : 0.4684307

Evaluation test

n presences : 59

n absences : 61

AUC : 0.9736038

cor : 0.8742979

max TPR+TNR at : 0.2394595

***Athene noctua* - CCSM**

Var. importance: bio.11 18.6439081, bio.1 12.0165075, bio.3 10.9995750, bio.18 8.3642021, bio.8 8.3135295

Evaluation train

n presences : 241

n absences : 239

AUC : 1
cor : 0.9940568
max TPR+TNR at : 0.632494

Evaluation test
n presences : 59
n absences : 61
AUC : 0.9883301
cor : 0.9270336
max TPR+TNR at : 0.5241273

***Athene noctua* - CNRM**

Var. importance: bio.3 20.9184087, bio.11 15.6547577, bio.2 12.7514810, bio.1 10.9950320, bio.8 6.9049423

Evaluation train
n presences : 242
n absences : 238
AUC : 1
cor : 0.9969772
max TPR+TNR at : 0.7241992

Evaluation test
n presences : 58
n absences : 62
AUC : 0.987208
cor : 0.901498
max TPR+TNR at : 0.2855782

***Athene noctua* - FGOALS**

Var. importance: bio.11 24.514685, bio.1 12.708851, bio.3 9.306664, bio.13 8.081401, bio.8 5.454867

Evaluation train
n presences : 249
n absences : 231

AUC : 0.9967837

cor : 0.9782365

max TPR+TNR at : 0.7078737

Evaluation test

n presences : 51

n absences : 69

AUC : 0.9951691

cor : 0.9405124

max TPR+TNR at : 0.6601653

***Athene noctua* - GISS**

Var. importance: bio.3 31.9721083, bio.11 17.0371972, bio.12 9.1929154, bio.8 8.0229132, bio.1 7.0424751

Evaluation train

n presences : 251

n absences : 229

AUC : 0.9999478

cor : 0.9872221

max TPR+TNR at : 0.5743415

Evaluation test

n presences : 49

n absences : 71

AUC : 0.9945387

cor : 0.9381722

max TPR+TNR at : 0.3389205

***Athene noctua* - IPSL**

Var. importance: bio.6 15.2132793, bio.1 13.5194233, bio.11 12.1745212, bio.3 9.5410608, bio.7 8.3599807

Evaluation train

n presences : 235

n absences : 245

AUC : 1

cor : 0.9938195

max TPR+TNR at : 0.6890475

Evaluation test

n presences : 65

n absences : 55

AUC : 0.9946853

cor : 0.923719

max TPR+TNR at : 0.3675275

***Athene noctua* - MIROC**

Var. importance: bio.3 31.0141007, bio.11 14.1930145, bio.1 9.6536193, bio.8 6.4603731, bio.15 5.3650119

Evaluation train

n presences : 239

n absences : 241

AUC : 1

cor : 0.9939859

max TPR+TNR at : 0.5315395

Evaluation test

n presences : 61

n absences : 59

AUC : 0.9894415

cor : 0.9131714

max TPR+TNR at : 0.6348607

***Athene noctua* - MPI**

Var. importance: bio.3 30.9601747, bio.11 19.5312486, bio.8 7.6808741, bio.2 5.4325166, bio.12 5.1016200

Evaluation train

n presences   : 247

n absences   : 233

AUC   : 1

cor   : 0.9962273

max TPR+TNR at : 0.7009319

Evaluation test

n presences   : 53

n absences   : 67

AUC   : 0.9811321

cor   : 0.8922638

max TPR+TNR at : 0.6930431

***Athene noctua* - MRI**

Var. importance: bio.1 23.1416300, bio.11 14.6345929, bio.14 7.3415449, bio.17 7.0933998, bio.12 6.9708652

Evaluation train

n presences   : 237

n absences   : 243

AUC   : 1

cor   : 0.9909717

max TPR+TNR at : 0.4629204

Evaluation test

n presences   : 63

n absences   : 57

AUC   : 0.9955444

cor   : 0.9368047

max TPR+TNR at : 0.623228

***Perdix perdix* - CCSM**

Var. importance: bio.1 16.322308, bio.10 15.862596, bio.19 14.951666, bio.15 11.302701, bio.17 9.556328

Evaluation train

n presences : 245

n absences : 237

AUC : 1

cor : 0.9988212

max TPR+TNR at : 0.7174145

Evaluation test

n presences : 55

n absences : 63

AUC : 0.9939394

cor : 0.9534171

max TPR+TNR at : 0.7064351

***Perdix perdix* – CNRM**

Var. importance: bio.15 39.7405088, bio.10 8.0932517, bio.5 7.8907712, bio.1 7.4604754, bio.3 6.5049240

Evaluation train

n presences : 248

n absences : 233

AUC : 0.9997923

cor : 0.9872866

max TPR+TNR at : 0.5755065

Evaluation test

n presences : 52

n absences : 67

AUC : 0.999713

cor : 0.9791666

max TPR+TNR at : 0.5144568

***Perdix perdix* - FGOALS**

Var. importance: bio.15 4.831420e+01, bio.17 1.464201e+01, bio.5 1.011540e+01, bio.8 8.076905e+00, bio.18 4.047200e+00

Evaluation train
n presences : 236
n absences : 244
AUC : 1
cor : 0.9997509
max TPR+TNR at : 0.9241894

Evaluation test
n presences : 64
n absences : 56
AUC : 0.9866071
cor : 0.9216309
max TPR+TNR at : 0.740028

Perdix perdix **- GISS**

Var. importance: bio.17 19.058030107, bio.19 16.706378930, bio.10 16.270777901, bio.3 9.066544159, bio.5 7.027111292

Evaluation train
n presences : 242
n absences : 238
AUC : 1
cor : 0.9977341
max TPR+TNR at : 0.6105089

Evaluation test
n presences : 58
n absences : 62
AUC : 0.9810901
cor : 0.8995122
max TPR+TNR at : 0.2507997

Perdix perdix **- IPSL**

Var. importance: bio.19 44.63000749, bio.10 17.12395841, bio.5 7.81144083, bio.15 6.23561744, bio.17 5.35595937

Evaluation train
n presences : 239
n absences : 241
AUC : 1
cor : 0.9955846
max TPR+TNR at : 0.6327539

Evaluation test
n presences : 61
n absences : 59
AUC : 0.9905529
cor : 0.9624265
max TPR+TNR at : 0.3374962

***Perdix perdix* - MIROC**

Var. importance: bio.19 38.5821392, bio.10 20.2412279, bio.17 14.7364038, bio.5 8.1896501, bio.1 3.7271071

Evaluation train
n presences : 243
n absences : 238
AUC : 1
cor : 0.9957908
max TPR+TNR at : 0.521158

Evaluation test
n presences : 57
n absences : 62
AUC : 0.9966044
cor : 0.9388863
max TPR+TNR at : 0.1468404

***Perdix perdix* - MPI**

Var. importance: bio.10 18.93415102, bio.19 16.32492366, bio.15 13.67923633, bio.8 10.38049365, bio.3 8.57528937

Evaluation train
n presences : 236
n absences : 245
AUC : 0.9999654
cor : 0.9928401
max TPR+TNR at : 0.3980764

Evaluation test
n presences : 64
n absences : 55
AUC : 0.9928977
cor : 0.9421292
max TPR+TNR at : 0.6264473

***Perdix perdix* - MRI**

Var. importance: bio.15 32.38157929, bio.1 11.83441988, bio.17 9.79539528, bio.5 9.70230038, bio.19 9.39490661

Evaluation train
n presences : 237
n absences : 243
AUC : 0.999618
cor : 0.9836852
max TPR+TNR at : 0.3259169

Evaluation test
N presences : 63
n absences : 57
AUC : 0.9966583
cor : 0.9496465
max TPR+TNR at : 0.6055084

Supplementary Data S3 - Values of the cells of the LGM ensembles corresponding to fossil occurrences. The values range from 0 (0 out of 8 models predict the occurrence of the species in that cell) to 8 (all 8 models predict the occurrence of the species in that cell).

Pyrrhocorax graculus: 6 6 5 7 5 7 6 5 6 6 6 7 7 4 6 6 5 7 5 6 6 6 7 6 8 7 4 4 7 7 6 6 4 6 6 7 7 5 6 8 6 7 8 8 4 6 8 8

Bubo scandiacus (breeding range): 0 0 0 0 0 0 1 1 0 1 1 0 0 0 1 0 1 0 0 0 1 1 0 0 0

Bubo scandiacus (wintering range): 0 2 2 3 0 2 1 6 0 2 4 2 1 1 5 3 2 4 2 4 1 1 3 2 1

Athene noctua: 7 8 8 8 8 8 8 7 8

Perdix perdix: 4 2 4 3 4 2 2 2 3 2 1 4 3 4 2 4 3 7 4 6 2 6 3 4 6 4 6 4 6 6 2 2 6 3 3

Crex crex (breeding range): 3 4 7 3

Coturnix coturnix (breeding range): 7 5 7 6 6 5 8 7 7 8 8 8 8 7 7 8 6 7

Supplementary Figure S4 - Elevation map of Europe. The legend is reported on the right side. The numbers represent the height in meters. The map was created with R, version 4.0.3 (https://www.R-project.org/).

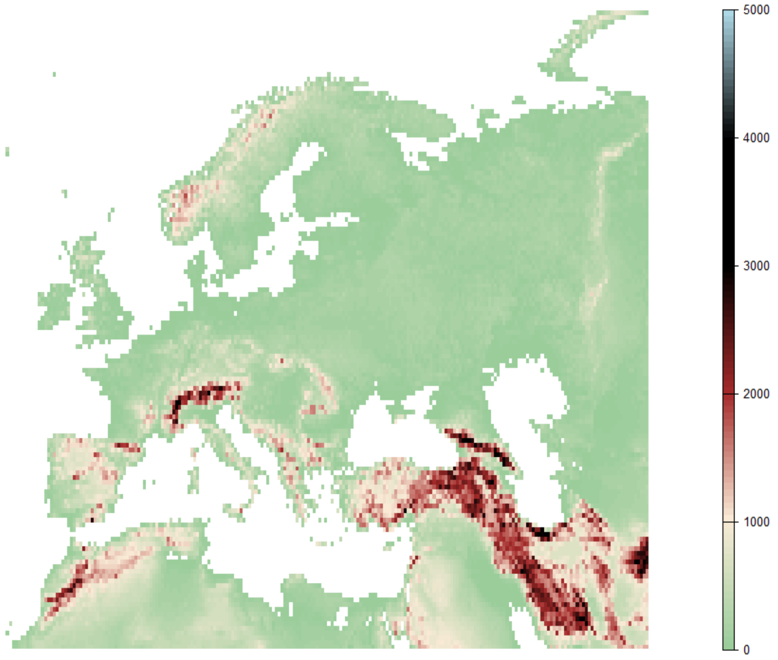

Supplementary Figure S5 - Elevation map of Africa. The legend is reported on the right side. The numbers represent the height in meters. The map was created with R, version 4.0.3 (https://www.R-project.org/).

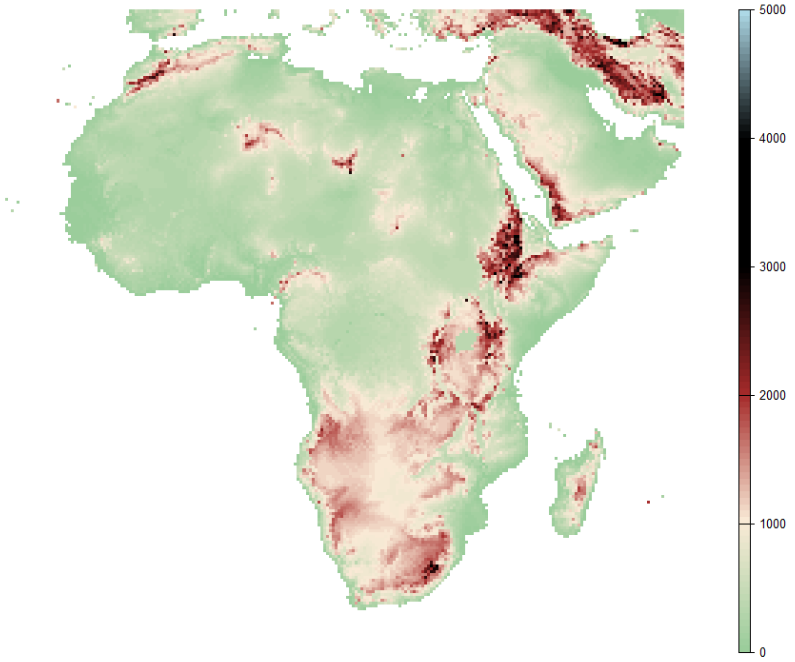

Supplementary Table S6 - List of the MIS 2 fossil occurrences of the six species in the Western Palearctic. In the tables are reported the names of the fossil localities, the layers of provenance, the geographic coordinates and the main bibliographic references

Pyrrhocorax graculus

Site name and layer	Latitude	Longitude	Bibliographic reference
Brillenhöhle (Baden-Württemberg) Layer V	48.403935	9.780276	[3]
Brillenhöhle (Baden-Württemberg) Layer VI	48.403935	9.780276	[3]
Sesselfelsgrotte (Bayern) Layer C	48.935528	11.789297	[4]
Sandalja II Layer E	44.888780	13.883458	[3]
Grotte de la Vache (Ariége) Layer IV	42.821281	1.587741	[3, 4, 10]
Grotte des Harpons (Haute-Garonne) Layer D	43.234823	0.663978	[3]
Abri de Campalou (Drôme) Layer 2	45.068396	5.254231	[3]
Abri de Campalou (Drôme) Layer 3	45.068396	5.254231	[3]
Grotte d'Embulla (Pyrénées-Orientale) Layer 1	42.581468	2.416360	[3]
Isturitz (Pyrénées-Atlantiques) Layer Salle de St Martin	43.352904	-1.206127	[3, 10]
Isturitz (Pyrénées-Atlantiques) Layer Salle de St Martin, La Grande Salle Niveau II	43.352904	-1.206127	[3, 10]
La Madeleine (Dordogne)	44.966876	1.036410	[3]
La Madeleine (Dordogne) Layer 9	44.966876	1.036410	[3]
La Madeleine (Dordogne) Layer 13	44.966876	1.036410	[3]
Laugerie Haute Est (Dordogne) Layer 2-20	44.952249	1.001764	[3]
Piage (Lot) Layer c-E	44.8044	1.3897	[3]
Rond-du Barry (Haute-Loire) Layer D	45.071203	3.859404	[3]
Rond-du Barry (Haute-Loire) Layer E	45.071203	3.859404	[3]
Rond-du Barry (Haute-Loire) Layer F	45.071203	3.859404	[3]
Salpêtriére à Remoulins (Gard) Layer C 4	43.938924	4.564192	[3]
Salpêtriére à Remoulins (Gard) Layer C 5	43.938924	4.564192	[3]
Salpêtriére à Remoulins (Gard) Layer D	43.938924	4.564192	[3]
Salpêtriére à Remoulins (Gard) Layer "ensemble i"	43.938924	4.564192	[3]
Salpêtriére à Remoulins (Gard) Layer 5	43.938924	4.564192	[3]
Trois Frères (Ariège)	43.032113	1.211584	[3, 10]
Combe Sauniére 1 (Dordogne) Layer IV	45.238806	0.873718	[3]
Grotte du Bison (Yonne) Layer C	47.601199	3.756028	[3]
Grotte du Bison (Yonne) Layer C	47.5911	3.7651	[3]
Aurensan inférieure (Hautes-Pyrénées)	43.617109	-0.203386	[3]
Baume de Gigny (Jura) Layer VI	46.469155	5.475109	[3]
Bois du Cantet (Hautes-Pyrénées) Layer Secteur 1	43.059066	0.295046	[3]
Bois du Cantet (Hautes-Pyrénées) Layer Secteur 2	43.059066	0.295046	[3]
Bois-de-Brousses (Hérault) Layer 1A	43.553084	3.256859	[3]
Bois-de-Brousses (Hérault) Layer 2B	43.553084	3.256859	[3]
Cauna de Belvis (Aude) Layer C6	42.849496	2.076573	[3]
Grotte Gazel (Aude) Layer 7	43.323602	2.419950	[3]
Grotte Gazel (Aude) Layer C.7	43.323602	2.419950	[10]
Grotte Jean-Pierre No. 1 (Savoie) Layer 9	45.500497	5.843928	[3, 4]
Grotta della Serratura (Salerno) Layer 9	39.998057	15.371955	[4]
Arene Candide (Liguria) Layer P1	44.165450	8.330301	[3]
Arene Candide (Liguria) Layer P3	44.165450	8.330301	[3]
Arene Candide (Liguria) Layer P4	44.165450	8.330301	[3]
Arene Candide (Liguria) Layer P7	44.165450	8.330301	[3]

Arene Candide (Liguria) Layer P8	44.165450	8.330301	[3]
Riparo Salvini (Lazio)	41.287284	13.251601	[3, 4]
Cueva de Abauntz (Navarra) Layer e	43.013899	-1.641201	[4]
Aitzbitarte IV (Guipuzcoa)	43.262471	-1.895824	[3]
Cova des Cendres (Alicante) Layer XI	38.720295	0.182650	[3, 4]
Cueva de Ambrosio (Almeria) Layer II (=Nivel 5)	37.821993	-2.099180	[3, 4]
Ekain (Guipuzcoa) Layer IV	43.236567	-2.275960	[3]
Ekain (Guipuzcoa) Layer Via	43.236567	-2.275960	[3]
Ekain (Guipuzcoa) Layer VII	43.236567	-2.275960	[3]
Ekain (Guipuzcoa) Layer VIII	43.236567	-2.275960	[3]
Erralla Cave (Guipuzcoa) Layer Level IV (spit 11-12)	43.2089	-2.1819	[3]
Arbreda (Gerona) Layer D	42.161581	2.746261	[3, 4; 11]
Arbreda (Gerona) Layer C	42.161581	2.746261	[3, 4, 11]
Arbreda (Gerona) Layer 13-16 (Probably Layers B, C)	42.161581	2.746261	[3, 4]
Urtiaga (Guipuzcoa) Layer F-G	43.295176	-2.353938	[3]
Urtiaga (Guipuzcoa) Layer I	43.295176	-2.353938	[3]
Combe-Cullier (Lot) Layer 5	44.8456	1.5664	[3]
Grotte d'Ebbou (Ardèche)	44.5100	4.0800	[3]
Laroque II (Hérault) Layer b	43.9167	3.7333	[3]
Tournal (Aude) a	43.3167	2.8833	[3]
Tournal (Aude) b	43.3167	2.8833	[3]
Tournal (Aude) c	43.3167	2.8833	[3]
Tournal (Aude) Layer "Couche à sagaies"	43.3167	2.8833	[3]
Covolo di Trene (Vicenza)	45.5214	11.4744	[3]
Palidoro (Lazio) Layer 1	41.9333	12.1833	[3]
Palidoro (Lazio) Layer 3	41.9333	12.1833	[3]
Palidoro (Lazio) Layer 6	41.9333	12.1833	[3]
Palidoro (Lazio) Layer 8	41.9333	12.1833	[3]
Cueva de el Parco (Lérida) Layer N.IV Int. Cueva	41.9086	0.9419	[4]
Cueva de el Parco (Lérida) Layer N.VI Int. Cueva	41.9086	0.9419	[4]
Cueva de el Parco (Lérida) Layer N.X. Int. Cueva	41.9086	0.9419	[4]
Roccia San Sebastiano Layer c2	41.1357	13.8799	[12, 13]
Roccia San Sebastiano Layer e	41.1357	13.8799	[12, 13]
Roccia San Sebastiano Layers cuts 1-6	41.1357	13.8799	[12, 13]
Grotta del Pozzo	41.9723	13.6717	[12]
Coulet des Roches (Vaucluse) c	44.0775	5.426308	[14]
Aitzbitarte III Layer III	43.2706	-1.8905	[15]
Cava a Filo	44.44285	11.37982	[16]
Lapa do Picareiro Layer T	39.5305	-8.6520	[17, 18, 19, 20]
Grotta di Ortucchio	41.9561	13.6467	[21]

Bubo scandiacus

Grotte de la Vache (Ariége) Layer IV	42.821281	1.587741	[3, 4]
Isturitz (Pyrénées-Atlantiques) Layer la Grand Salle Niveau I	43.352904	-1.206127	[3]
Isturitz (Pyrénées-Atlantiques) Layer la Grand Salle Niveau II	43.352904	-1.206127	[3]
La Madeleine (Dordogne)	44.966876	1.036410	[3]
Piage (Lot) Layer c-E	44.8044	1.3897	[3, 22]
Rond-du Barry (Haute-Loire) Layer E	45.071203	3.859404	[3]
Trois Frères (Ariège)	43.032113	1.211584	[3]

Combe Sauniére 1 (Dordogne) Layer IV	45.238806	0.873718	3, 4
Abri Dufaure (Landes) Layer 6	43.535847	-1.072403	3, 4
Baume de Gigny (Jura) Layer VI	46.469155	5.475109	3
Bois-Ragot, Gouex (Vienne) Layer BR5b	46.365774	0.688330	3, 4
Bois-Ragot, Gouex (Vienne) Layer BR4b	46.365774	0.688330	3, 4
Bois-de-Brousses (Hérault) Layer 1a	43.553084	3.256859	3
Arene Candide (Liguria) Layer P1	44.165450	8.330301	3
Arene Candide (Liguria) Layer P4	44.165450	8.330301	3
Arene Candide (Liguria) Layer P7	44.165450	8.330301	3
Arene Candide (Liguria) Layer P9	44.165450	8.330301	3
Pekárna (Moravia)	49.2628	16.7823	3, 23
Flageolet II (Dordogne) Layer IX	44.8500	1.0833	3
Fontarnaud (Girondet)	44.7500	-0.1667	3
Grotte du Placard (Charente) Layer "Brèche"	45.6833	0.4167	3
Grottes de Jaurias (Gironde)	44.8261	-0.2887	3, 22
Covolo di Trene (Vicenza)	45.5214	11.4744	3
Coulet des Roches b	44.0775	5.426308	14
Grotte de Lourdes (Hautes-Garonne) coll. Harle (Middle and Upper Magd layer)	43.091004	-0.045874	22
Plantade (Tarn-et-Garonne) corniche sup., excav. Brun, Middle and Upper Magdalenian layers	45.5833	2.6000	22
Gandil (Tarn-et-Garonne) c.20, c.25 et c.23, excav. Ladier	44.0500	1.6667	22
Saint-Germain-la-Riviere (Gironde) ens. sup., excav. Trecolle	44.9500	-0.3167	22
Taillis des Coteaux (Vienne) Layer IIIa, excav. Primault	46.53	0.85	22, 24
Taillis des Coteaux (Vienne) Layer IIg, excav. Primault	46.53	0.85	22
Roc de Marcamps 2 (Gironde) excav. Lenoir	45.0322	-0.5020	22

Athene noctua

Rond-du Barry (Haute-Loire) Layer E	45.071203	3.859404	3
Combe Sauniére 1 (Dordogne) Layer IV	45.238806	0.873718	3, 4
Arene Candide (Liguria) Layer P7	44.165450	8.330301	3
Arene Candide (Liguria) Layer P9	44.165450	8.330301	3
Riparo Salvini (Lazio)	41.287284	13.251601	3, 4
Cueva de Ambrosio (Almeria) Layer I (=Nivel 6.1-6.3)	37.821993	-2.099180	3, 4
Cueva de Ambrosio (Almeria) Layer II (=Nivel 5)	37.821993	-2.099180	3, 4
Palidoro (Lazio) Layer 8	41.9333	12.1833	3
Tossal de la Roca (Alicante) Layer III	38.7902	-0.2810	3
Lapa do Picareiro Layer T	39.5305	-8.6520	17, 19, 20
Grotta di Ortucchio	41.9561	13.6467	21

Perdix perdix

Grotte du Bois Laiterie (Namur) Layer TT	50.3500	4.8500	4
Grotte du Bois Laiterie (Namur) Layer YSS	50.3500	4.8500	4
Grotte du Bois Laiterie (Namur) Layer BSC	50.3500	4.8500	4
Geissenklösterle (Baden-Württemberg) Layer AH Io	48.398207	9.772127	3, 4
Grotte de la Vache (Ariége) Layer IV	42.821281	1.587741	3, 4
Abri de Campalou (Drôme) Layer 2	45.068396	5.254231	3, 4
Grotte d'Embulla (Pyrénées-Orientale) Layer 1	42.581468	2.416360	3
La Madeleine (Dordogne)	44.966876	1.036410	3
Pont d'Ambon (Dordogne) Layer 4	45.3014	0.5382	3
Rond-du Barry (Haute-Loire) Layer D	45.071203	3.859404	3

Rond-du Barry (Haute-Loire) Layer E	45.071203	3.859404	[3]
Rond-du Barry (Haute-Loire) Layer E 3	45.071203	3.859404	[3]
Rond-du Barry (Haute-Loire) Layer F 2	45.071203	3.859404	[3]
Salpêtriére à Remoulins (Gard) Layer D	43.938924	4.564192	[3]
Trois Frères (Ariège)	43.032113	1.211584	[3]
Combe Sauniére 1 (Dordogne) Layer IV	45.238806	0.873718	[3, 4]
Aurensan inférieure (Hautes-Pyrénées)	43.617109	-0.203386	[3]
Baume de Gigny (Jura) Layer V	46.469155	5.475109	[3]
Bois du Cantet (Hautes-Pyrénées) Layer Secteur 1	43.059066	0.295046	[3]
Bois-de-Brousses (Hérault) Layer 2B	43.553084	3.256859	[3]
Flageolet I (Dordogne) Layer V	44.848653	1.068722	[3]
Grotte Gazel (Aude) Layer 7	43.323602	2.419950	[3]
Grotte Jean-Pierre No. 1 (Savoie) Layer 9	45.500497	5.843928	[3, 4]
Grotta della Serratura (Salerno) Layer 9	39.998057	15.371955	[4]
Arene Candide (Liguria) Layer P1	44.165450	8.330301	[3]
Arene Candide (Liguria) Layer P3	44.165450	8.330301	[3]
Arene Candide (Liguria) Layer P4	44.165450	8.330301	[3]
Arene Candide (Liguria) Layer P7	44.165450	8.330301	[3]
Arene Candide (Liguria) Layer P8	44.165450	8.330301	[3]
Arene Candide (Liguria) Layer P9	44.165450	8.330301	[3]
Riparo Salvini (Lazio)	41.287284	13.251601	[3, 4, 21]
Cueva de Abauntz (Navarra) Layer e	43.013899	-1.641201	[4]
Arbreda (Gerona) Layer 13-16 (Probably Layers B, C)	42.161581	2.746261	[3, 4]
Arbreda (Gerona) 17 (Probably Layer D)	42.161581	2.746261	[3, 4]
Urtiaga (Guipuzcoa) Layer F-G	43.295176	-2.353938	[3]
Tournal (Aude) a	43.3167	2.8833	[3]
Palidoro (Lazio) Layer 1	41.9333	12.1833	[3]
Palidoro (Lazio) Layer 3	41.9333	12.1833	[3]
Palidoro (Lazio) Layer 6	41.9333	12.1833	[3]
Palidoro (Lazio) Layer 7	41.9333	12.1833	[3]
Palidoro (Lazio) Layer 8	41.9333	12.1833	[3]
Roc de la Melca (Gerona)	42.4200	1.5500	[3]
Grotta di Ortucchio (Aquila)	41.9561	13.6467	[4, 21]
Zupanov spodmol Layer C-D	45.7450	14.1142	[4]
Roccia San Sebastiano Layer c2	41.1357	13.8799	[12, 13]
Roccia San Sebastiano Layer e	41.1357	13.8799	[12, 13]
Roccia San Sebastiano Layers cuts 1-6	41.1357	13.8799	[12, 13]
Grotta del Pozzo	41.9723	13.6717	[12]
Aitzbitarte III Layers III	43.2706	-1.8905	[15]
Pekárna Cave	49.2628	16.7823	[23]
Cava a Filo	44.44285	11.37982	[16]
Lapa do Picareiro Layer T	39.5305	-8.6520	[17, 18, 19, 20]
Lapa do Picareiro Layer U	39.5305	-8.6520	[17, 18, 19, 20]

Crex crex

Bois-Ragot (Vienne) Layer BR5b	46.365774	0.688330	[3, 4]
Arene Candide (Liguria) Layer P1	44.165450	8.330301	[3]
Arene Candide (Liguria) Layer P9	44.165450	8.330301	[3]
Temnata Cave Layer 3 c/d	43.174288	24.072201	[3, 4]

Biśnik Cave (Czestochowa uplands) Layer Complex II	50.4264	19.8317	[4, 25]

Coturnix coturnix

Sandalja II Layer E	44.888780	13.883458	[3]
Abri de Campalou (Drôme) Layer 3	45.068396	5.254231	[3]
Pont d'Ambon (Dordogne) Layer 4	45.3014	0.5382	[3]
Rond-du Barry (Haute-Loire) Layer D	45.071203	3.859404	[3]
Rond-du Barry (Haute-Loire) Layer E	45.071203	3.859404	[3]
Rond-du Barry (Haute-Loire) Layer E 3	45.071203	3.859404	[3]
Rond-du Barry (Haute-Loire) Layer F	45.071203	3.859404	[3]
Salpêtriére à Remoulins (Gard) Layer C3	43.938924	4.564192	[3]
Salpêtriére à Remoulins (Gard) Layer "ensemble i"	43.938924	4.564192	[3]
Aurensan inférieure (Hautes-Pyrénées)	43.617109	-0.203386	[3]
Baume de Gigny (Jura) Layer V	46.469155	5.475109	[3]
Grotta della Serratura (Salerno) Layer 9	39.998057	15.371955	[4]
Arene Candide (Liguria) Layer P1	44.165450	8.330301	[3]
Arene Candide (Liguria) Layer P4	44.165450	8.330301	[3]
Arene Candide (Liguria) Layer P7	44.165450	8.330301	[3]
Arene Candide (Liguria) Layer P8	44.165450	8.330301	[3]
Arene Candide (Liguria) Layer P9	44.165450	8.330301	[3]
Riparo Salvini (Lazio)	41.287284	13.251601	[3, 4]
Cueva de Nerja (Malaga) Layer 13	36.761594	-3.846283	[3]
Arbreda (Gerona) Layer D	42.161581	2.746261	[3, 4, 11]
Arbreda (Gerona) Layer C	42.161581	2.746261	[3, 4, 11]
Palidoro (Lazio) Layer 8	41.9333	12.1833	[3]
Roc de la Melca (Gerona)	42.4200	1.5500	[3]
Ohalo 2 (Galilee)	32.722093	35.572143	[4]
Roccia San Sebastiano Layer c2	41.1357	13.8799	[12, 13]
Roccia San Sebastiano Layer e	41.1357	13.8799	[12, 13]
Roccia San Sebastiano Layers cuts 1-6	41.1357	13.8799	[12, 13]
Aitzbitarte III Layers III	43.2706	-1.8905	[15]
Grotta di Ortucchio	41.9561	13.6467	[21]

Supplementary References

1. BirdLife International. Pyrrhocorax graculus. *The IUCN Red List of Threatened Species 2016: e.T22705921A87386602*. https://dx.doi.org/10.2305/IUCN.UK.2016-3.RLTS.T22705921A87386602.en (2016).
2. Cramp, S. *The complete birds of the Western Palearctic on CD-ROM* (Optimedia, Oxford University Press, 1998).
3. Tyrberg, T. *Pleistocene birds of the Palearctic: a catalogue.* (Publications of the Nuttall Ornithological Club No. 27, 1998).
4. Tyrberg, T. *Pleistocene birds of the Palaearctic.* http://web. telia.com/~u11502098/pleistocene.pdf (2008).
5. BirdLife International. Bubo scandiacus. *The IUCN Red List of Threatened Species 2020: e.T22689055A181375387*. https://dx.doi.org/10.2305/IUCN.UK.2020-3.RLTS.T22689055A181375387.en (2020).
6. BirdLife International. Athene noctua. *The IUCN Red List of Threatened Species 2019: e.T22689328A155470112*. https://dx.doi.org/10.2305/IUCN.UK.2019-3.RLTS.T22689328A155470112.en (2019).
7. BirdLife International. Perdix perdix. *The IUCN Red List of Threatened Species 2016: e.T22678911A85929015*. https://dx.doi.org/10.2305/IUCN.UK.2016-3.RLTS.T22678911A85929015.en (2016).
8. BirdLife International. Crex crex. *The IUCN Red List of Threatened Species 2016: e.T22692543A86147127*. https://dx.doi.org/10.2305/IUCN.UK.2016-3.RLTS.T22692543A86147127.en (2016).
9. BirdLife International. Coturnix coturnix. *The IUCN Red List of Threatened Species 2018: e.T22678944A131904485*. https://dx.doi.org/10.2305/IUCN.UK.2018-2.RLTS.T22678944A131904485.en (2018).
10. Laroulandie, V. Alpine chough *Pyrrhocorax graculus* from Pleistocene sites between Pyrenees and Alps: natural versus cultural assemblages in *Birds in Archaeology: Proceedings of the 6th Meeting of the ICAZ Bird Working Group in Groningen (23.8-27.8. 2008)* (eds Prummel, W., Zeiler, J. T., Brinkhuizen, D. C), 219-232 (Barkhuis, 2010).
11. Lloveras, L. *et al*. The role of birds in Upper Palaeolithic sites: Zooarchaeological and taphonomic analysis of the avian remains from Arbreda Cave (Serinyà, northeast Iberia). *Quat. Int*., 10.1016/j.quaint.2020.10.022 (2020).
12. Gala, M., Fiore, I. & Tagliacozzo, A. Human exploitation of avifauna during the Italian Middle and Upper Paleolithic in *Palaeolithic Italy: Advanced studies on early human adaptations in the Apennine peninsula* (eds. Borgia, V. & Cristiani, E.), 183-217 (Sidestone press, 2018).
13. Ruiu, F. D. *et al*. The fauna from the Gravettian levels of Roccia San Sebastiano Cave (Mondragone, Caserta, Italy) in *Proceedings of the General Session of the 11th International Council for Archaeozoology Conference (Paris, 23-28 August 2010)* (eds Lefèvre, C.), 99-111 (Archaeopress, 2012).
14. Crégut-Bonnoure, E. *et al*. The karst of the Vaucluse, an exceptional record for the Last Glacial Maximum (LGM) and the Late-glacial period palaeoenvironment of southeastern France. *Quat. Int*. **339**, 41-61 (2014).
15. Sánchez-Marco, A. Aves del Pleistoceno Superior de Aitzbitarte III (País Vasco) in *Ocupaciones Humanas En Aitzbitarte III (País Vasco) 33.600-18.400 BP (Zona de entrada a la cueva)* (eds. Altuna, J., Mariezkurrena, K. & Ríos, J.) 493-505 (Eusko Jaurlaritzaren Argitalpen Zerbitzu Nagusia, 2011).
16. Paronuzzi, P. Nota preliminare sulla sequenza UMG di ex Cava a Filo (Croara, BO): gli aspetti stratigraficosedimentari, paleontologici e antropici alla luce delle ultime indagini (2006–2016). *Memorie dell'Istituto Italiano di Speleologia* **2**, 131-144 (2018).

17. Estraviz López, D. *Quaternary fossil vertebrates from continental Portugal: Paleobiodiversity, revision of specimens and new localities*. PhD Dissertation, Universidade de Évora (2019).
18. Figueiredo, S. M. D. (2010). *A Avifauna Plistocénica de Portugal: especificidades evolutivas, anatómicas eo seu contexto paleontológico, geológico e arqueológico*. PhD Dissertation, Universidade de Salamanca (2010).
19. Pimenta, C., Moreno-García, M. & Lourenço, A. O registo ornito-arqueológico em Portugal: inventários, comentários e mapas. *Revista Portuguesa de Arqueologia* **18**, 289-312 (2015).
20. Hockett, B. & Haws, J. Continuity in animal resource diversity in the Late Pleistocene human diet of Central Portugal. *Before Farming* **2**, 1-14 (2009).
21. Gala, M. & Tagliacozzo, A. (2010). The avifauna from Late Glacial archaeological sites in Italy: a tentative synthesis in *Birds in Archaeology: Proceedings of the 6th Meeting of the ICAZ Bird Working Group in Groningen (23.8-27.8. 2008)* (eds Prummel, W., Zeiler, J. T., Brinkhuizen, D. C), 205-218 (Barkhuis, 2010).
22. Laroulandie, V. Hunting fast-moving, low-turnover small game: The status of the snowy owl (*Bubo scandiacus*) in the Magdalenian. *Quat. Int*. **414**, 174-197 (2016).
23. Musil, R. Phenological analysis of the Last Glacial vertebrates from the territory of Moravia (the Czech Republic)-continuity and change in faunistic communities. *Fossil Imprint* **74**, 199-236 (2018).
24. Rambaud, D., Laroulandie, V., Primault, J. & Bearez, P. Les poissons et les oiseaux du Taillis des Coteaux (Antigny, Vienne), niveaux magdaléniens: origine naturelle ou culturel in *Taphonomie des petits vertébrés: Référentiels actuels et transferts aux fossiles* (eds Laroulandie V., Mallye J. B. et Denys C.), 167-179 (Archaeopress, 2011).
25. Tomek, T., Bocheński, Z. M., Socha, P. & Stefaniak, K. Continuous 300,000-year fossil record: changes in the ornithofauna of Biśnik Cave, Poland. *Palaeontol. Electron*. **15**, 1-20 (2012).
